# Neuroprotective Effect of Caffeine in Alzheimer’s Disease

**DOI:** 10.3390/molecules27123737

**Published:** 2022-06-10

**Authors:** Y Mukish M Yelanchezian, Henry J. Waldvogel, Richard L. M. Faull, Andrea Kwakowsky

**Affiliations:** 1 Centre for Brain Research, Department of Anatomy and Medical Imaging, Faculty of Medical and Health Science, University of Auckland, Auckland 1023, New Zealand; ymye313@aucklanduni.ac.nz (Y.M.M.Y.); h.waldvogel@auckland.ac.nz (H.J.W.); rlm.faull@auckland.ac.nz (R.L.M.F.); 2 Pharmacology and Therapeutics, School of Medicine, Galway Neuroscience Centre, National University of Ireland Galway, H91 W5P7 Galway, Ireland

**Keywords:** caffeine, coffee, cognition, Alzheimer’s disease, dementia

## Abstract

Alzheimer’s disease (AD) is the leading cause of dementia, predicted to be the most significant health burden of the 21st century, with an estimated 131.5 million dementia patients by the year 2050. This review aims to provide an overview of the effect of caffeine on AD and cognition by summarizing relevant research conducted on this topic. We searched the Web of Science core collection and PubMed for studies related to the effect of caffeine on AD and cognition using title search terms: caffeine; coffee; Alzheimer’s; cognition. There is suggestive evidence from clinical studies that caffeine is neuroprotective against dementia and possibly AD (20 out of 30 studies support this), but further studies, such as the “ideal” study proposed in this review, are required to prove this link. Clinical studies also indicate that caffeine is a cognitive normalizer and not a cognitive enhancer. Furthermore, clinical studies suggest the neuroprotective effect of caffeine might be confounded by gender. There is robust evidence based on in vivo and in vitro studies that caffeine has neuroprotective properties in AD animal models (21 out of 22 studies support this), but further studies are needed to identify the mechanistic pathways mediating these effects.

## 1. Introduction

Alzheimer’s disease (AD) is reported to be the leading cause of dementia and the significant healthcare burden of the 21st century [1]. In 2015, over 46 million people were reported to be living with dementia (costing US 818 billion), a figure projected to reach 131.5 million dementia patients by the year 2050 [2]. Developed countries with an aging population are expected to be worse hit by this burden. Typical clinical presentation of dementia includes memory impairment and executive function decline that interferes with daily activities making the elderly less independent and forcing them to engage with support services [1]. Atypical presentation of dementia consists of a more pronounced memory deficit causing language, visual, and executive problems [1]. Atypical dementia is more common in early-onset dementia, which is reported to have a strong genetic component [1].

The pathophysiology of AD is based on the accumulation of abnormally folded *Aβ* and Tau proteins in amyloid plaques and neuronal tangles that contribute to neurodegeneration in patients’ brains [3]. Much of this evidence comes from studying familial AD, where there exist mutations in APP genes, which alter the action of *γ*-secretases that cleaves Amyloid Precursor Protein (APP), causing an accumulation and aggregation of *Aβ* peptide [4]. Hyperphosphorylated Tau (PTau) protein, another prerequisite for AD diagnosis, accumulates intracellularly and fibrillates into paired helical filaments that form neurofibrillary tangles [5]. It has been proposed that PTau can further accelerate *Aβ* dysfunction [5]. A significant genetic factor for AD is APOE mutations, with the lifetime risk of AD being 50% for homozygous APOE4 carriers and 20–30% for APOE3 and APOE4 heterozygous carriers [6]. However, even without an APOE mutation at 85 yr of age, there is an 11–15% risk of developing AD, indicating there might be a significant environmental component to AD [6]. Recent evidence has suggested many lifestyle factors: diabetes, diet, socioeconomic status (SES), education, physical and mental activity, depression, tobacco use, and alcohol intake may affect the chance of developing AD and dementia [7]. The Rotterdam study even demonstrated that eliminating the seven most hazardous risk factors could reduce the incidence of dementia by 30% [8]. In the absence of a definitive clinical treatment currently, eliminating these modifiable risk factors is our best tool for reducing the burden of dementia and AD.

Coffee, the most heavily consumed caffeinated beverage, has been a popular research topic in AD, with several epidemiological studies positing its neuroprotective effect [9]. Coffee comprises a few independently neuroprotective components: caffeine, chlorogenic acid, caffeic acid, and trigonelline [10]. Chlorogenic acid has been shown to reduce blood-brain barrier (BBB) damage and improve neuronal differentiation in mice [11]. Caffeic acid and phytochemicals in coffee act as antioxidative and anti-inflammatory substances, thereby helping reduce cognitive decline [12]. Trigonelline from coffee beans has been shown to alleviate neuronal loss by reducing oxidative stress, astrocyte activity, and neuroinflammation while preserving mitochondrial integrity [13]. The neuroprotective properties of coffee have been heavily linked to its high caffeine content, but this has been difficult to demonstrate independently through epidemiological studies due to the confounding effect of other components in caffeinated beverages [9].

Caffeine is a natural trimethyl xanthine alkaloid in which the three methyl groups are located at positions 1, 3, and 7 (1,3,7-Trimethylxanthine) [14]. Caffeine, a key psychoactive ingredient in coffee, is a short-acting neurostimulator with known neuromodulator effects on the brain by inhibiting phosphodiesterase, mobilizing intracellular calcium, antagonism of adenosine receptors, and modulation of GABA receptor function [15]. Rodent studies have also reported caffeine can inhibit amylogenic-*Aβ* protein production and improve cognition in rodent AD models [16]. Caffeine has high oral bioavailability, with 99% of caffeine being absorbed from the gastrointestinal (GI) tract into the bloodstream 45 min after ingestion. A peak plasma concentration of 1–10 μM (0.25–2 mg/L) reached 15–120 min post oral ingestion from a cup of coffee in humans [17].

However, the results from previous studies are controversial, with some reporting caffeine to be neuroprotective while others report no effects or even detrimental effects on cognition. Therefore, this study aims to clarify the impact of caffeine on cognition and AD by reviewing all the relevant research published on this topic.

## 2. Methods

In the present review, we searched the Web of Science core collection and PubMed for studies related to the effect of caffeine and cognition. We used the title search terms caffeine, coffee, Alzheimer’s, cognition and excluded all reviews.

We did not use the search term tea because it returned many results related to the neuroprotective action of herbal tea, which was not associated with the action of caffeine, the main interest of this review. Additionally, Eskelinen et al. reported that tea drinking in midlife was not correlated with AD development [18]. They posited that this could be because of tea’s comparatively lower caffeine content than coffee [18]. However, this could be biased as the study recruited fewer tea drinkers than coffee drinkers [18]. The average caffeine content per drink was 60.4 +/− 21.8 mg for instant coffee (14-fold range), 80.1 +/− 19.2 mg for brewed coffee (2.8-fold range), and 28.8 +/− 13.7 mg for tea (5.5-fold coverage) [19].

Through our review we identified 20 clinical studies that supported the notion that caffeine/coffee exerts a neuroprotective effect on cognition against dementia and AD ([18,20,21,22,23,24,25,26,27,28,29,30,31,32,33,34,35,36,37,38]) (Table 1). Our review of the literature also found 10 clinical studies that did not support the notion of neuroprotective effect of caffeine/coffee [39,40,41,42,43,44,45,46,47,48] (Table 1). We also reviewed 21 animal model studies that showed caffeine as neuroprotective [49,50,51,52,53,54,55,56,57,58,59,60,61,62,63,64,65,66,67,68,69] (Table 2) and used them to explain caffeine’s expected mechanism of action. We also identified one in vitro and in vivo study that did not link caffeine and cognition [70] (Table 2).

## 3. Clinical Studies Investigating the Relationship between Caffeine and Cognition

### 3.1. Longitudinal Epidemiological Studies

Cao et al., gathered two cohorts of 124 participants (65–88 yrs old) and measured their plasma caffeine concentration as well as their initial neurological status (based on clinical history, clinical dementia rating (CDR), Multi-Mental State evaluation (MMSE), psychiatric evaluation, Three-trial Fluid Object Memory Evaluation (TFOME), Hopkins’s verbal Learning Test Revised (HVLTR), MRI-volumetric protocol and National Alzheimer’s Disease and Clinical Centre (NACC) protocol [20]. Participants were grouped into three categories of cognitive functions: Normal (M), Mild cognitive impairment (MCI), and Dementia (DEM); then, the participants were followed up for a period of 2–4 yrs, and their cognition was reassessed (same protocol) [20]. This study found that participants who demonstrated a cognitive decline from initial MCI to DEM had a significantly lower plasma caffeine concentration (by 51%) than participants who maintained the level of cognitive impairment (stable MCI) [20]. Furthermore, none of the subjects with a critical plasma caffeine concentration (>1200 ng/mL) converted to DEM, and half of stable MCI had plasma concentrations greater than this critical value [20]. Fredholm et al. 1999, found that this plasma concentration of coffee (1200 ng/mL or 6 μM) is the typical plasma caffeine concentration several hours after ingestion of 1–2 cups of coffee after decreasing from a peak of 10 μM–20 μM an hour after coffee ingestion as reported by Culm-Medrek et al., 2005 [71,72]. Cao et al., also measured 11 cytokines and found that 3 (GCSF, IL-10, and IL-6) were lower in participants who had a cognitive decline from initial MCI to DEM [20]. The main strength of this study is that it uses plasma caffeine concentration which is a more objective measure than using a recall dietary survey of caffeine intake as used in other studies [20]. However, this approach is also associated with a few limitations, like only one measurement of plasma caffeine levels was obtained; thus, it does not allow detection of a change in caffeine habit [20]. Also, the study did not account for other confounding variables, i.e., lifestyle choices. The follow-up period of 2–4 yrs may be too short to determine if caffeine-cognition has a true causal relationship [20].

Another study recruited 1445 cognitively normal subjects screened with: the Babcock Story Recall Test (BRST), Activities of Daily Living (ADL), and MMSE, then grouped them into two groups: rarely consumed coffee (<1 cup/day) and habitual moderate coffee drinker (1–2 cups/day) based on Food Frequency Questionnaire (FFQ) results; subjects were followed up for a 3.5 yrs median and incidence of developing MCI based on MMSE, GDS was recorded [21]. Solfrizzi and colleagues found that habitual moderate coffee drinkers (HR:0.31, 95% CI:0.13–0.75) had a lower incidence of MCI than rare coffee drinkers (HR:0.47, 95% CI:0.211–1.02); this minor difference by hazard ratios of 0.16 indicates that there is only a slight decrease in risk of dementia among coffee consumers [21]. The study also found that those who altered their coffee habit had an increased risk of MCI than those with a constant coffee habit: increased by >1 cup (HR:1.8, 95% CI:1.11–2.92), decreased by <1 cup (HR:2.17, 95% CI:1.16–4.08) this shows that there is almost doubling of dementia risk when there is a change in the coffee habit [21]. Furthermore, high coffee habit > 2 cups/day had no correlation with MCI incidence compared to those who rarely consumed coffee (HR:0.26, 95% CI:0.03–2.11), as shown by the large CI [21]. A significant strength of this study is that it showed the effect of changing coffee habits on the risk of developing MCI [21]. However, this study has a few limitations like recall bias as a coffee habit was based on FFQ, underestimation of caffeine intake as it does not include other sources of caffeine (tea, chocolate,) and a short follow-up period [21].

As part of a larger study, Driscoll et al., recruited 6467 participants from Women Health Initiative (WHI) Hormonal therapy RCT trial and determined their self-reported caffeine intake at enrolment using FFQ [22]. Then the subject’s cognitive function was screened annually for ten years or less over the telephone using a 100-point Modified Mini-Mental State (3MS) exam and a 40-point Telephone Interview for Cognitive Status-modified (TICSM); those showing cognitive decline were followed up by board-certified psychiatrists using the Dementia Questionnaire (DQ) to verify MCI [22]. Then proportional hazard regression (HR) was performed to assess the risk of developing MCI based on their baseline caffeine intake while adjusting for risk factors: such as hormone therapy, age, race, education, BMI, sleep quality index, depression, hypertension, diabetes, history of cardiovascular disease (CVD), smoking and alcohol consumption [22]. Driscoll et al., found that women consuming above the median (175 mg/d) levels of caffeine (mean intake = 261 mg for this group) had only a small effect on lowering the risk of developing dementia by 26% or any cognitive impairment (HR = 0.74, 95% CI:0.60–0.91) compared to those consuming below median levels (mean intake = 64 mg for this group) [22]. This study also reported that a cup of 8-ounce black coffee contained 95 mg of caffeine; thus, the median caffeine intake is equivalent to 1.8 cups of black coffee [22]. A significant strength of this study is that the subjects were extensively screened and characterized through a detailed prospective follow-up study, which helped adjust for confounding variables and a short lag between screens which helped provide a more sensitive record of MCI onset [22]. However, this study has limited generalizability as only older post-menopausal women who tended to be better educated were included in the study [22].

Paganini-Hill et al., recruited 587 subjects >90 yrs old (mean = 93 yrs) who showed no signs of dementia at enrolment, and their lifestyle factors data (smoking, alcohol, caffeine intake, vitamin supplement, and exercise) was also collected at enrolment, and 20 yrs previously from the Leisure World Cohort health survey (1981–1985) [23]. Then the participants were followed up for 36 months, and their cognitive status was determined by trained health professionals using a battery of neuropsychological tests: neurological exam, MMSE, informant questionnaire, DQ, and CASI-short [23]. This study found that those who consumed >200 mg/day of caffeine had a significantly lower risk of about 34% (HR = 0.66, *p* < 0.05) of dementia than those who consumed <50 mg/day of caffeine by using Cox regression analysis [23]. A strength of this study is that it prospectively studied cognitively normal individuals and routinely examined their cognitive status with a short lag time in-between, which allowed early identification of cognitive decline and dementia [23]. However, this study has some severe limitations as it only assessed lifestyle factors from 2-time points 20 yrs apart. It did not account for habit changes, and the study subjects were also predominantly moderately affluent Caucasians, limiting the study’s generalizability [23].

In a large-scale study, 13,137 cognitively healthy subjects were recruited (>65 yrs) from the Ohsaki Cohort study 2006, FFQ assessed their coffee intake, and other lifestyle factors were also simultaneously collected and adjusted for: baseline age, BMI, green tea consumption, education, CVD history, fractures, stroke, diabetes, smoking, alcohol intake and social support [24]. The subjects were followed up for 5.7 yrs, and incidents of dementia were calculated based on data from the Long-Term Care Insurance database [24]. A multivariate analysis found that coffee consumption and incidence of dementia for the categories: never, occasionally, 1–2 cups/day, ≥3 cups/day had a hazard ratio of 1.00, 0.73 (95% CI: 0.62–0.82), 0.72 (95% CI: 0.61–0.84) and 0.82 (95% CI: 0.65–1.02), respectively. These findings indicated that 1–2 cup and ≥3 cups was moderately neuroprotective, reducing by 28% and 18%, respectively [24]. This protective effect of caffeine was more significant among women, non-smokers, and non-drinkers groups than in the studies’ general population. Compared to the previous study, a substantial benefit of this study was its very large sample size and multivariate adjustment for confounders, which included social support [24]. However, this study did have a few limitations in assessing coffee intake: it did not measure coffee intake in midlife, did not assess if there was a change of habit after baseline, and did not differentiate between decaffeinated caffeinated coffee [24]. This study also may have suffered from some reverse causality due to a lack of data sensitivity as they did not exclude those who showed a cognitive decline at baseline but were not certified disabled [24].

Haller et al. recruited 45 elderly controls and 18 MCI who were chronic coffee consumers (1–3 cups/day) [25]. They were followed up for 18 months, and their cognitive function was assessed (MMSE), which was used to group them into Stable-controls cognitive (24-sCON), mild cognitive impairment (18-MCI), and deteriorating-controls (21-dCON) [25]. The participants were put on caffeine detox for 18 h and then given 200 mg caffeine or a placebo 30 min before being subjected to an n-back task (established WT fMRI test) [25]. The MR data were analyzed with: a hypothesis-driven general linear model (GLM) analysis of task-related activation, tensorial induced component analysis (TICA) of functional connectivity, arterial spin labeling (ASL) perfusion, Gray matter voxel-based morphometry (VBM), and white matter DTI tract-based special statistics (TBSS) [25]. Haller et al., found no difference in working memory (assessed by fMRI n-back tasks) performance between sCON and dCON, while MCI was less accurate and slower (*p* < 0.05) [25]. Furthermore, the dCON group also had a less pronounced acute caffeine-induced brain activation, which was restricted to the right hemisphere (*p* < 0.05) and reduced caffeine-induced Default Mode Network deactivation compared to sCON (*p* < 0.01) [25]. This decreased sensitivity of caffeine effects in dCON is in line with the idea caffeine is a cognitive normalizer instead of a cognitive enhancer, and complex fMRI patterns are possibly due to existing functional changes despite behavioral pattern maintenance [25]. Strengths of this study were the exclusion of potential confounding alteration in GM by VBM and WM by DTI TBSS; and using ASL to measure brain perfusion, which confirmed that even though there was a generalized 25–30% decrease in CBF, there was not any localized change in CBF, ensuring that fMRI changes are not due to global CBF differences. This study also has several limitations, such as a small sample size and the inability to comment on long-term changes in brain activation patterns induced by caffeine [25]. Then there is also the possibility of non-excludable de-novo brain pathologies during follow-up [25].

In another study, Haller et al., recruited 145 cognitively stable elders screened by cognitive test (MMSE, HAD, LIDA) and used a self-administered questionnaire to assess their coffee, chocolate, and wine consumption [26]. They were followed up for 3 yrs, during which MRI imaging and two neuropsychological examinations were administered testing: attention, working, digit episodic, executive, language, visual, phenomics verbal fluency, praxis ideomotor, reflexive, and constructive memory [26]. These data were used to group participants into stable-cognitive (52-sCON), intermediate-cognitive (62-iCON) and deteriorating-cognitive (32-dCON). The MRI data were analyzed by: whole-brain VBM, ASL, diffusion tensor imaging TBSS, and GM region of interest (ROI) analysis [26]. This study found that moderate coffee consumers are less likely to be categorized as dCON (OR_adjusted_: 0.447, 95% CI:0.210–0.952, *p* = 0.037) [26]. Moreover, MRI imaging found a negative correlation between VBM and caffeine only for sCON, notably in the WM (left parietal and right frontal), indicating fewer WM lesions and increased cerebral blood flow, but there was no association among iCON and dCON [26]. This positive relationship between cognition and caffeine only being present in sCON further supports the notion that caffeine is a cognitive normalizer and not a cognitive enhancer [26]. The strength of this study includes longitudinal follow-up and a lack of health-related confounders [26]. However, there were also a few limitations, like the study subject being adults who lack vascular pathology, limiting the study’s generalizability among the general population with CVD pathology [26].

West et al., recruited 638 elderly (+65 yrs) subjects with Type 2 diabetes (T2D) from the Israel Diabetes and Cognitive Decline study (IDCD). The subject’s caffeine intake was recorded using FFQ [33]. Their cognition was measured using a neuropsychological test battery that looked at four factors: episodic memory, executive function, semantic categorization, and working memory [33]. A further subgroup of subjects (185) was randomly selected and subjected to MRI imaging to estimate WM and GM volumes [33]. West et al., using linear regression adjusting for cognitive-related covariates (SES, diet, cardiovascular, thyroid, and type of T2D), found that higher caffeine intake was related to better overall cognition (*p* = 0.018), working (*p* = 0.002), executive function (*p* = 0.047), semantic memory (*p* = 0.026). This effect on cognition was amplified in the older group (above median) compared to the younger [33]. The MRI imaging also found that higher caffeine intake resulted in higher GM volume (B = 0.198, *p* = 0.033) [33]. Which indicates reduced neuronal death and a possible mechanism of neuroprotective action of caffeine [33]. The strength of this study is that it uses a large study sample size and a unique study population (T2D) while adjusting for potential confounding [33]. However, this also acts as a limitation as it makes it difficult to generalize this study’s findings to the non-T2D population [33].

Vercambre et al., recruited 2475 elderly (+65 yrs) women from the Women’s Antioxidant Cardiovascular Study (WACS), and their caffeine intake at baseline was assessed by 116 item-food frequency questionnaires [27]. Then their global cognition was assessed using four Telephone Interview of Cognitive Status (TICS) at 2-year intervals. Vercambre et al., reported consumption of caffeinated coffee was correlated with significantly slower rates of cognitive decline (*p* = 0.05) but not for other caffeinated products, i.e., tea, chocolate, and cola [27]. The rate of difference between the highest and lowest quantile of caffeine intake (>371 and <30 mg/day) and cognitive decline was equivalent to that of 7 yrs apart in age (*p* = 0.006) [27]. The strengths of this study were that it was a longitudinal study, adjusting for confounders, and included a large sample size [27]. However, there were also several limitations: using a self-reported questionnaire to assess caffeine intake increases the risk of recall bias, the study only looked at caffeine intake at baseline, which might not reflect long term use, and this study focused on caffeine as the sole active component in coffee, but other studies have reported that polyphenols in coffee might exert an independent protective effect against cognitive decline [27].

Eskelinen et al., randomly recruited 1409 individuals (followed up for 21 (median = 4.9) years) from the FINMONICA study (1972, 1977, 1982, 1987), which examined lifestyle, diet, BMI, BP, health status, and serum cholesterol through a self-administered questionnaire [18]. During the follow-up in 1998, the previous survey method was readministered, and ApoE genotyping and cognitive screening using MMSE, DSM-IV, and NINCDS-ADRDA [18]. The study reported that coffee drinkers in midlife had a markedly lower risk of developing dementia and AD even after adjustment [18]. The lowest risk of dementia (OR: 0.34, 95% CI = 0.16–0.73) and AD (OR: 0.38, 95% CI = 0.17–0.89) was found among chronic coffee consumers (3–5 cups/day) at midlife indication there is about a 66–62% reduction in risk [18]. Strengths of this study include a long follow-up period, validated protective effects of caffeine against cognitive decline even after adjusting for hyperlipidemia, and looking at coffee consumption in midlife using a validated questionnaire [18]. Limitations of this study include the possibility of recall bias due to the self-reported questionnaire used and a sample size that is too small to detect possible dose-response effects [18].

Ritchie et al., recruited 4197 women and 2820 men, and their coffee consumption (questions in the standardized interview) as well confounding factors (age, gender, BMI, education, diet, lifestyle, medical history, alcohol, and tobacco intake) were assessed at baseline, and a 2- and 4-year follow-up [28]. The subject’s cognition was assessed with the MMSE, Benton Visual retention test (BVRT), Issacs set test (IST), and DSM-IV at baseline and subsequent follow-ups [28]. Ritchie et al., multivariate mixed models and multivariate-adjusted logistic regression indicated that women with higher caffeine consumption (>3 cups/day) showed less decline in verbal retrieval (OR = 0.67, CI = 0.53–0.85) and visuospatial memory (OR = 0.82, CI = 0.65–1.03) over 4 yrs than women consuming one cup/day or less [28]. This protective effect was enhanced with age by 43%, 65–74 yrs (OR = 0.73, CI = 0.53–1.02) and 80+ yrs (OR = 0.3, CI = 0.14–0.63), indicating that the neuroprotective effect of caffeine is significantly confounded by age in women [28]. There was no relation between cognitive decline and caffeine intake in men [28]. The strengths of this study are a longitudinal design, a large sample size, and validated methods of measuring cognition [28]. Limitations of this study are selective attrition could have promoted a healthy survivor effect among subjects, and a follow-up period longer than 4 yrs may be necessary to evaluate risk and benefit adequately [28].

Gelber et al., recruited 3734 Japanese Americans, their coffee intake was assessed through 24-h dietary recall, and their cognition was screened using 100-point Cognitive Abilities Screening Instrument (CASI) [39]. A subgroup of 418 participants was subjected to autopsies where their brains were studied for neuropathological lesions (Alzheimer’s lesions), microvascular ischemic lesions, neocortical Lewis bodies, hippocampal sclerosis, and generalized brain atrophy) [39]. The study reported no association between coffee intake in midlife and risk of cognitive impairment [39]. However, it did find that the highest quartile of coffee intake (≥411.10 mg/day) was less likely than the lowest quartile of coffee drinkers (≤137.0 mg/day) to have any type of brain lesion in postmortem examination (OR: 0.45, 95% CI = 0.23–0.89, *p* = trend 0.04) [39]. The strengths of this study are it measures coffee intake in midlife, has a large sample size, and is the first study to investigate the link between coffee intake with postmortem lesions [39]. The limitation of this study is that it only includes men from a particular ethnic and social group which limits the generalizability of the findings [39].

Araujo et al., recruited 2914 participants (59 ± 7.2 yrs.) and at baseline, performed coffee consumption analysis (FFQ), brain MRI and cognitive test battery (Letter digit substitution task (LDST), Stroop test, Word fluency test (WFT), 15-word learning test (WLT) and Purdue pegboard (PBB) [40]. Then the subjects were followed up for 5 yrs, and the baseline analysis was repeated [40]. The cross-sectional segment of this study at baseline reported higher caffeine intake was associated with minor reduction in prevalence of lacunar infarcts (OR per cup = 0.88, 95% CI = 0.79–0.98), smaller hippocampus volume (diff size = −0.01, 95% CI = −0.02–0.00), small improvement in cognitive performance in: LDST (difference = 1.13, 95% CI = 0.39–1.88), WFT (difference = 0.74, 95% CI = 0.04–1.45), Stroop test (1.182, 95% CI = 0.23–3.41), and worse cognitive performance in WLT (−0.38, 95% CI = 0.74–0.02) but this minor reduction of risk might not result in noticeable changes by the person [40]. Furthermore, after the five-year repeat cognitive assessment, the association between higher caffeine intake and improved cognition was not found. The study hypothesized that this discrepancy could be because caffeine is a short-term neurostimulator and long-term exposure to caffeine causes the body to build up a tolerance, and thus the effect of caffeine wanes [40]. However, these relationships were not found when followed up longitudinally [40]. Strengths of the study include having a population-based design with large sample size and using brain MRI to measure cognitive function in the same population. The limitation of the study is that it did not look for a change in the coffee habit during the follow-up period [40].

Mirza et al., recruited 4368 subjects from the Rotterdam study; their coffee consumption was measured as part of a home interview, covariate data (BMI, age, health, smoking, alcohol, lifestyle) was gathered from the Rotterdam Study, and cognition was assessed through MSSE, geriatric mental schedule (GMS), and Cambridge examination for mental disorders among the elderly (CAM-DEX) [41]. The study data was stratified into short follow-up (0–4 yrs) and long follow-up (>4 yrs) till 21 yrs [41]. Mirza et al., reported that during short follow-up, they found those who consumed >3 cups/day had a 30% lower risk of developing dementia (HR = 0.70, 95% CI = 0.51–0.96) than those who drank <1 cup/day [41]. However, this relationship did not extend to the long follow-up [41]. The study reported this could be subject to confounding as the subject reported that chronic coffee consumers are also more likely to have a poor lifestyle with a high lipid diet which is a risk factor for dementia [41]. Strengths of this study include having a large sample size, long follow-up with stratification, and an intensive dementia case verification protocol [41]. Limitation of this study: coffee intake was based on dietary recall increasing the risk of recall bias, and other confounding factors were not accounted for [41].

Arab et al., recruited 4809 participants (>65 yrs) from the cardiovascular health study and followed them up for a median of 7.9 yrs [29]. The subject’s intake of caffeinated beverages was assessed using FFQ, and their cognition was measured annually using Modified mini-mental state examinations [29]. The study adjusted for confounding (age, education, SES, depression, APOE genotype, medical history, smoking) and used linear mixed models to analyze the data [29]. This study found that in fully adjusted models, the intake of coffee and tea modestly reduced rates of cognitive decline in some but not all women, and there was no dose-effect relationship among the women [29]. No consistent effect was identified for men [29]. The strengths of this study are that it has a large follow-up period and uses a validated measure of cognition [29]. The limitation of this study is that FFQ did not specify the amount of beverage but only its frequency which reduces the accuracy of the dose-response findings [29].

Fisher et al., recruited 2622 (75+ yrs.) participants from the German study on aging, cognition, and dementia (AgeCoDE) whose food intake was measured using a single-food questionnaire (wine, coffee, green tea, olive oil, fresh fruits, vegetable, and red meats) and their incidence of dementia and AD assessed through (CERAD and SIDAM) over 10 yrs. was recorded [42]. The data was then analyzed using multivariate-adjusted joint modeling considering gender and Apolipoprotein E4 (APOE e4) [42]. Fisher et al., did not find any statistically significant association between coffee consumption and the incidence of dementia/AD [42]. The strength of this study was the inclusion of dropout time to allow an unbiased account of cognitive decline in the presence of missing data [42]. The limitation of this study was that dietary recall was only done at baseline, and they did not measure for change in the dietary habit over follow-up [42].

Santos et al., included 648 participants (≥65 yrs), and assessed their baseline (1999–2003) caffeine intake using FFQ and cognition using MMSE [30]. They were then followed up, and their cognition was assessed again using MMSE (2005–2008) [30]. The data was then adjusted for confounding factors: age, education, gender, smoking, alcohol, BMI, hypertension, and diabetes [30]. Santos et al., reported that those with the third quartile of caffeine intake (>62 mg/day) had a significantly lower risk of cognitive decline by 51% (RR = 0.49, 95% CI = 0.24–0.97) compared to those with the first quartile of caffeine intake (<22 mg/day) among women only [30]. This reduction is expected to result in noticeable benefits for women. No statistically significant relationship between caffeine intake and cognition was found among men [30]. The limitation of this study was that it did not account for incomplete follow-up as only 58.2% of the initial cohort completed the study; therefore, there could be selection bias that limits the study’s generalizability [30].

### 3.2. Cross-Sectional Studies

Cornelis et al., recruited 445,786 participants (37–73 yrs) from 22 biobank centers across the UK [45]. The subjects filled out an extensive questionnaire that contained caffeinated beverage intake (used to calculate average daily caffeine), medical history, lifestyle, and diet [45]. Participants’ cognition was screened for prospective memory (PM), pairs matching (Pairs), Symbol Digit Substitution (SDS), fluid intelligence (FI), and reaction time (RT) using a computerized cognitive function test [45]. The data was then analyzed using multivariate analysis to identify interactions between coffee, tea, and genetic-based caffeine metabolism score (CMS) on cognitive function [45]. Cornelis et al., reported that coffee intake (≥1 cup) significantly decreased reaction time, pairs matching, Trail making test B, and symbol digit substitution. No, statistically significant relationship was identified between cognitive function and CMS × tea, CMS × coffee, and CMS × caffeine [45]. The strength of this study was its large sample size and its ability to adjust for genetic caffeine metabolism. Limitations include the possibility of unmeasured confounding, and the study sample also suffers from “healthy volunteer’ selection bias and may not represent the wider population [45].

Another study by Cornelis et al., recruited 434,900 participants (37–73 yrs) from 22 biobank centers across the UK [46]. The subjects filled out an extensive questionnaire that contained medical history, lifestyle, and diet [46]. Recent caffeine intake (last hour) was recorded during the physical assessment, where participants completed at least one out of four self-administered cognitive function tests: prospective memory (PM), pairs matching (Pairs), fluid intelligence (FI), and reaction time (RT) [46]. The data was then analyzed using multivariate analysis to identify interactions between recent caffeine intake, genetic-based caffeine metabolism score (CMS), and cognitive function [46]. Cornelis et al. [46] reported that among white participants recent coffee consumption was correlated with higher RT performance but worse FI, Pairs, and PM (*p* ≤ 0.004) [46]. Among non-white participants similar associations were found FI (*p* = 0.09), Pairs (*p* = 0.03), and PM (*p* = 0.34) [46]. The limitations of this study are that yes/no self-reported recent caffeine consumption, which suffers from recall bias, was used instead of a biomarker, and information regarding caffeine source, amount, or preparation was not considered [46].

Ritchie et al., recruited 1193 elderly (+65 yrs), including those with depressive symptomology and T2D [43]. The subject’s caffeine intake was recorded at baseline during the interview, along with their cognition (MMSE), serum glucose, and B-amyloid levels (known memory confounders) were also recorded [43]. Higher caffeine intake was linked to a significant decrease in incidental diabetes in men (HR:0.64, 95% CI:0.42–0.97) and a significant increase in incidental diabetes risk in women (HR:1.51, 95% CI:1.08–2.1), no statistically significant association was found between caffeine and depression or *Aβ* levels [43]. The study also did not find that caffeine was neuroprotective against dementia among women [43]. The study found no evidence that decreased risk of dementia among heavy caffeine-consuming women was confounded by diabetes or depression [43]. A limitation of this study, like many others, is that it assumes that caffeine is the only neuroprotective substance in tea or coffee, but Alves et al., have reported on the estrogenic properties of coffee through its high isoflavone content, which could independently exert neuroprotective properties [43,73].

Kim et al., recruited 411 subjects and screened them using the CERAD-K neuropsychological examination into cognitive normal (CN = 282) and MCI = 129 [31]. The participant’s coffee consumption (current and lifetime) was grouped into the low coffee intake (<2) and high coffee intake (≥2). Then the subjects underwent PET and MRI scans to measure cerebral *Aβ* deposition, AD-CM, AD-CT, and WMH [31]. This study found that higher coffee intake was significantly associated with lower *Aβ* positivity than low coffee consumption, even after adjusting for confounding factors [31]. However, current nor lifetime coffee intake was associated with hypometabolism, AD-signature region, and WMH volume [31]. This absence in a change of WM volume contrasted with MRI studies by Ritchie et al., and Haller et al., who found caffeine decreased the amount of WM lesion/cranial volume in cognitively stable elders [26,35]. The strength of this study is that it is the first study to investigate the association between coffee intake and in vivo AD pathologies [31]. However, it also has a few limitations; since it is a cross-sectional study, it cannot establish a causal relationship between coffee and cognition. Since coffee intake was based on recall, there is an increased risk of recall error [31].

A cross-sectional study by Iranpour and colleagues recruited 1440 adults (>60 yrs) from the National Health and Nutrition Examination Survey (NHANES) [34]. Twenty-four-hour dietary recall data assessed the caffeine intake, and cognition was measured using CERAD and DSST. They also collected covariate data on age, sex, SES, lifestyle, BMI, and history of the disease. The study found that the highest quartile of caffeine intake was positively associated with better cognitive function in the crude model with a *p* < 0.05, which means the relationship is statistically significant [34]. After adjusting for confounding, the association was only marginally significant in the CERAD word recall test (*p* = 0.09), and this trend was enhanced among men (B = 0.001, *p* = 0.004) but not females (B = 0.00007, *p* = 0.89) [34]. This contrasts with the other three previous studies that found that the neuroprotective effect of caffeine was enhanced among women and not males [24,28,35]. This clearly, indicates that more studies are needed to comment on how the neuroprotective effect of caffeine is affected by gender. The strengths of this study are that it had a large sample size and accounted for various confounders [34]. However, this study also had a few limitations: being a cross-sectional study, it suffers from reverse causality and using dietary recall, which increases random measurement error [34].

Ritchie, et al., recruited 641 elderly (>65 yrs) persons and recorded their caffeine intake as well as confounding factors (SES, education, mobility, BMI, alcohol intake, smoking, disease) at baseline using a questionnaire [35]. Then the participants underwent MRI imaging to estimate white matter lesions (WML) and white matter volume (WM) volume [35]. Ritchie et al., found that the mean log-transformed WML/cranial volume ratio after adjusting for women who consumed more than three units of caffeine (−1.23, SD = 0.06) was significantly lower than women who consumed two to three units of caffeine (−1.04, SD = 0.04) or one unit or less (−1.04, SD = 0.07) [35]. The study also showed increased cerebral perfusion in chronic coffee consumers, indicating a possible neuroprotective mechanism of coffee [35]. However, this relationship did not extend to the male population, who had no statistically significant association between caffeine and WML [35]. A strength of this study was that it used large epidemiological data and considered multiple confounding factors [35]. However, the limitations of this study, like many done on this topic, was that it did not consider lifetime caffeine intake, and self-reported caffeine intake was used, which is prone to recall bias. Furthermore, MRI imaging was performed only once, so the study could not describe the neurological changes over time induced by caffeine intake [35].

Kyle et al., recruited 351 participants (64 yrs) born in 1936 and sat Moray House Test (MHT) [44]. The subjects underwent an interview with a health professional to extract information (medication, lifestyle, SES, gender, age, disease history) and had their cognition tested with MMSE [44]. The participant’s caffeine intake was also measured using a MONICA food frequency questionnaire [44]. The study found that caffeine intake was correlated with a slower digit symbol (F = 3.38, *p* < 0.02), but this was removed after accounting for SES [44]. Kyle et al., reported that once adjusted for confounding and SES, there was no evidence that caffeine affected cognition [44]. A limitation of this study is it used a self-reported questionnaire which increases the chance of recall bias, and the study also has a small sample size [44].

Dong et al., recruited 2513 participants (≥60 yrs) from the National Health and Nutrition Survey (NAHNES) [36]. Coffee and caffeine intake was recorded using two 24-h dietary recall questionnaires, and cognition was assessed using the CERAD test, animal fluency test, and DSST [36]. Dong et al., using binary logistic reasoning and restricted cubic spline models, reported that those with 226.4–495 g/day caffeine intake had a significantly better performance by 44% (OR = 0.56, 95% CI = 0.35–0.89) on DSST compared to those who reported no caffeine intake [36]. Furthermore, those who reported ≥384.8 g/day also had moderately better performance (OR = 0.68, 95% CI = 0.48–0.97) compared to the lowest quartile of caffeine intake and (OR = 0.62, 95% CI = 0.38–0.98) for CERAD [36]. A positive association was reported between caffeine/coffee intake and CERAD and DSST Score but no association between decaffeinated coffee and cognition [36]. The strength of this study was that it used a large nationally (USA) representative sample of older adults in the study [36]. However, as this is a cross-sectional study, it is more prone to reverse-causality and cannot confirm causality [36].

Additionally, a few cross-sectional studies did not directly examine the effect of caffeine on cognition. Al-Khateeb et al., studied the effect of serum copper/lipid on cognition and, incidentally, found that increased coffee intake demonstrated a 6.25-fold lower risk for cognitive decline [32]. Furthermore, Kim et al., and Hosking et al., examined the effect of caffeine on cognition as part of a specific diet and found that the coffee diet was correlated to worse cognitive performance [47,48]. However, these studies had a severe limitation as coffee was considered part of a diet that contained many components (i.e., high fat), posing a higher risk of cognitive decline [47,48].

### 3.3. Randomized Control Studies

Haler et al., performed a double-blind placebo-controlled fMRI study during an n-back working memory task in 17 individuals with MCI (70.7 ± 4.6 yrs) and 17 age-matched healthy individuals (HC) (68.3 ± 2.8 yrs) who were cognitively screened (MMSE, Hospital Anxiety and Depression Scale (HAD), Lawton’s instrumental daily activities (LIDA)) [38]. The MCI was then further described with neuropsychological examinations, which tested: (attention, working, digit, episodic, executive, language, visual, phenomics verbal fluency, praxis ideomotor, reflexive, and constructive) memory [38]. All subjects were chronic coffee consumers (1–3 cups/day) who were detoxed from caffeine for 18 h and given 200 mg of caffeine or placebo tablets 30 min before neuroimaging [38]. The scan data was assessed to measure behavioral data (SPSS statistics), General Linear Model (GLM) analysis of task-related activation, Tensorial-independent component analysis (TICA) analysis of functional connectivity, analysis of Atrial Spin Labelling (ASL) perfusion, grey matter voxel-based morphometry (VBM) analysis, and white matter microstructure Track based spatial statistics (TBSS) fractional anisotropy analysis [38]. The study reported that acute caffeine administration induced a more prefrontal activation in HC and a more diffuse posteromedial activation in MCI [38]. In MCI, TICA documented significant-caffeine related enhancement in the activation of the prefrontal cortex, supplementary motor areas, ventral premotor, parietal cortex, basal ganglia, and cerebellum compared to HC [38]. This suggests the posterior displacement of working memory-related brain activation patterns after caffeine administrations in MCI represents a compensatory mechanism to counterbalance frontal lobe dysfunction [38]. This also adds to the evidence that caffeine acts as a neuro normalizer instead of a neuroenhancer [38]. As previously suggested by longitudinal epidemiological studies, coffee might be a cognitive normalizer [25,26]. Additionally, West et al. found that caffeine’s effect on cognition was enhanced among the older group [33]. The absence of a significant difference in ASL signifies a neuronal difference rather than a purely perfusion difference. The exclusion of potentially confounding differences in VBM and TBSS analysis between HC and MCI reduces confounding due to differences in Grey-matter densities and white matter microstructure [38]. The strength of this study is that it uses both task-related model-driven and independent component data-driven analysis of fMRI while controlling for the direct vasoconstrictive effect of caffeine [38]. An obvious limitation of this study is the inability to comment on the long-term effects of caffeine, like stabilization of the Blood-Brain Barrier (BBB) [38].

Lin et al., recruited 20 healthy (good sleep patterns, no substance use, young (18–35), BMI (18–25)) male participants who were habitual coffee drinkers [37]. They were placed in a 9-day ambulatory phase during which ten were placed on (3 × 150 mg/day caffeine), and the other ten were given a placebo (3 × 150 mg/day mannitol) [37]. Then on the 10th day, subjects were woken at normal time and subjected to N-back task MRI imaging and EEG 12.75 h. after awakening and 5.5 h. after the last caffeine treatment [37]. The imaging data were analyzed for Grey Matter Volume (GMV) and cerebral blood flow (CBF) [37]. In this study, higher caffeine intake was associated with reduced GMV in the medial temporal lobe compared to placebo, even after adjusting for increased CBF induced by caffeine [37]. Caffeine treatment was also associated with poor working memory but not sleep quality [37]. The strength of this study is that, being an RCT and it managed to significantly reduce environmental confounding factors [37]. This study has some limitations, such as the small sample size and that the data might be affected by genetic caffeine insensitivity [37].

**Table 1:** Overview of clinical studies that investigates the relationship of caffeine and cognition in dementia.

Study	Study Design	Participants			Treatment			Main Outcomes
		Population Groups	Size	Age (yrs.)	Caffeine	Cognition		
						Follow Up	Tests	
[20] (Cao et al., 2012)	Longitudinal epidemiological study	Initial normal remained Normal during follow up (*n* = 60) Initial normal but declined to MCI during follow up (*n* = 9) Initial MCI and maintained MCI (*n* = 21) Initial MCI declined to DEM (*n* = 11) Initial DEM maintained DEM (*n* = 23)	124 subjects’ total Tampa cohort (*n* = 81) Miami cohort (*n* = 43)	65–88 yrs	Baseline Plasma caffeine concentration measured	Between 2–4 yrs. Average 2 ½–3 yrs	clinical history psychiatric evaluation MRI CDR MMSE TFOME HVLTR NACC protocol tests	subjects with cognitive decline during follow up (MCI > DEM), had significantly lower baseline plasma caffeine concentration than participants who maintained their level of cognitive impairment (stable MCI) a critical baseline plasma concentration of 1200 ng/mL was identified out of 11 cytokines measured, 3 (GCSF, IL-10, and IL-6) were lower in participants who experienced cognitive decline from initial MCI to DEM
[21] (Solfrizzi et al., 2015)	Longitudinal epidemiological study	rarely consumed coffee (0–1 cup/day), (*n* = 886) moderate levels of coffee consumers (1–2 cups/day), (*n* = 409) higher level of coffee consumers (>2 cups/day), (*n* = 150)	1445 cognitively normal at baseline subjects	65–84 yrs	FFQ at 1st = 1992–1993 2nd = 1995–1996	Median 3.5 yrs	BRST ADL MMSE	habitual moderate coffee drinkers had a lower risk of developing MCI than those who rarely drank coffee those who altered their coffee consumption habit had an increased risk of MCI than those with a constant coffee habit there was no MCI incidence correlation between those with higher levels of coffee consumption and those who rarely consumed coffee
[22] (Driscoll et al., 2016)	Longitudinal epidemiological study	<75 mg caffeine (*n* = 1293) 75–174 mg caffeine (*n* = 1608) 175–189 mg caffeine (*n* = 1784) ≥190 mg caffeine (*n* = 1737)	6467 only female subjects	65–80 yrs	FFQ at baseline	10 yrs. Or less Above or below median caffeine intake = 7.2 and 6.9 yrs. resp	3MS TICMS dementia questionnaire	median caffeine intake was 175 mg/daily women with above-median caffeine intake had a lower risk of developing dementia or any cognitive impairment compared to those consuming below median levels
[23] (Paganini-Hill et at., 2016)	Longitudinal epidemiological study	<50 mg caffeine 50–199 mg caffeine 200+ mg caffeine	587 cognitively normal at baseline subjects	90–103 yrs. Mean = 93 ± 2.6	Self-reported Questionnaire at enrolment and Leisure World Cohort health survey (1981–1985)	36 months.	Neurological exam MMSE informant questionnaire DQ CASI-short	those who consumed >200 mg/day of caffeine had a lower risk of dementia than those who consumed <50 mg/day of caffeine
[24] (Sugiyama et al., 2016)	Longitudinal epidemiological study	never coffee (*n* = 2048) occasionally (*n* = 4194) 1–2 cup/day (*n* = 5246) ≥3 cup/day (*n* = 1649)	13,137 non-cognitive disabled at baseline	>65 yrs	FFQ at baseline	5.7 yrs	Incidence of dementia reported to the insurance database	incidence of dementia was inversely associated to the consumption of coffee The inverse relationship was more remarkable among women, non-smokers, and non-drinkers
[25] (Haller et al., 2017)	Longitudinal epidemiological study	sCON, (*n* = 24) dCON, (*n* = 21) MCI, (*n* = 18)	45 elderly controls, 18 with MCI	sCON = 70.0 ± 4.3 dCON = 73.4 ± 5.9 MCI = 71.6 ± 4.7	Self-reported chronic coffee consumers (1–3 cups/day)	18 months	MMSE WM task in fMRI MR imaging	maintenance of working memory behavioral performance in dCON reduced caffeine-induced brain activation changes in dCON compared to sCON. caffeine is a cognitive normalizer, not cognitive enhancer
[26] (Haller et al., 2018)	Longitudinal epidemiological study	sCON (*n* = 52) iCON (*n* = 62) dCON (*n* = 32)	145 subjects	sCON = 73 ± 3 dCON = 74 ± 4 iCON = 73 ± 3	Substance questionnaire at baseline	3 yrs	MR Imaging Neuropsychological assessments MSSE HAD IADL	moderate coffee consumers are less likely to be categorized as dCON caffeine in sCON correlated to fewer WM lesions and increased cerebral blood flow but not in iCON and dCON caffeine is a cognitive normalizer, not cognitive enhancer
[27] (Vercambre et al., 2013)	Longitudinal epidemiological study	<30 mg/day caffeine 30–111 mg/day caffeine 112–203 mg/day caffeine 204–371 mg/day caffeine >371 mg/day caffeine	2475 cognitive healthy female health professional with CVD risk	65+ yrs	Willett semi-quantitative food questionnaire at baseline	5 yrs	TICS	rate of cogitative preservation between the highest and lowest quantile of caffeine intake was equivalent to that of 7 yrs. apart in age
[18] (Eskelinen et al., 2009)	Longitudinal epidemiological study	0–2 cups/day (*n* = 223) 3–5 cups/day (*n* = 641) >5 cups/day (*n* = 542)	1409 individuals	65–79 yrs	Survey questionnaire at baseline	21 yrs	MMSE DSM-IV NINCDS-ADRDA	coffee drinkers at midlife had a markedly lower risk of developing dementia and AD lowest risk of dementia and AD was found among chronic coffee consumers (3–5 cups/day) at midlife
[28] (Ritchie et al., 2007)	Longitudinal epidemiological study	0–1 unit/day (M = 27.4%, F = 24.6%) 1–2 unit/day (M = 32.4%, F = 31.5%) 2–3 unit/day (M = 27.0%, F = 27.5%) >3 unit/day (M = 13.2%, F = 16.4%)	7017 dementia free subjects. Men(M) = 2820. Female(F) = 4197	>65 yrs. M = average 73.6 ± 5.3 yrs. F = average 73.8 ± 5.2 yrs	Questions in the standardized interview by health professional at baseline	Average = 3.4 ± 0.67 yrs	BVRT IST DSM-IV MMSE	women with >3 cups/day showed less memory decline than women consuming ≤1 cup/day no relation between cognitive decline and caffeine intake in men
[39] (Gelber et al., 2011)	Longitudinal epidemiological study	0–115.5 mg/day (*n* = 707) >115.5–188.0 mg/day (*n* = 604) >188.0–277.5 mg/day (*n* = 784) >277.5–415.0 mg/day (*n* = 704) >415.0–2673 mg/day (*n* = 695)	3734 cognitive healthy Japanese American men. Autopsy sub-group (*n* = 418)	71–93 yrs. Mean = 52 yrs	24 h dietary recall questionnaire at entry (mid-life)	25 yrs	CASI	no association between midlife coffee intake and risk of cognitive impairment higher caffeine intake is associated with a lower incidence of any type of brain lesions at autopsy
[40] (Araujo et al., 2016)	Longitudinal epidemiological study with cross-sectional subgroup	0–1 cups/day >1–3 cups/day >3 cups/day	cognitive healthy subjects, 55% female. cross-sectional (*n* = 2914), longitudinal (*n* = 2454)	Mean = 59 ± 7.2 yrs	FFQ at baseline	5 yrs	MRI LDST Stroop test WFT WLT PBB	cross-sectionally reported higher caffeine intake was associated with a lower prevalence of lacunar infarcts, smaller hippocampus volume, and better cognitive performance. These relationships are not found longitudinally
[41] (Mirza et al., 2014)	Stratified Longitudinal epidemiological study	0–1 cups/day (*n* = 360) >1–3 cups/day (*n* = 1792) >3 cups/day (*n* = 3256)	Cognitively healthy subjects (0–4 yrs, *n* = 5408) (>4 yrs. *n* = 4935)	0–1 cups/day = 70.3(8.6) >1–3 cups/day = 69.5(7.8) >3 cups/day = 66.3(7.3)	Questionnaire baseline (*n* = 5408) Follow-up questionnaire (*n* = 4368)	Mean = 13.2 ± 5.4 yrs	MMSE GMS CAM-DEX	Short follow-up, >3 cups/day, had a lower risk of dementia than <1 cup/day This relationship is not found in long-follow-up
[29] (Arab et al., 2011)	Longitudinal epidemiological study	Tea < 5×/year (M = 26.5%, F = 21.1%) Tea 5–10×/year (M = 11.7%, F = 10.6%) Tea 1–3×/month (M = 18.6%, F = 18.4%) Tea 1–4×/week (M = 21.8%, F = 22.6%) Tea ≥ 5×/week (M = 21.3%, F = 27.2) coffee < 5×/year (M = 30.8%, F = 37.4%) coffee 5–10×/year (M = 6.7%, F = 6.2%) coffee 1–3×/month (M = 7.4%, F = 6.5%) coffee 1–4×/week (M = 10.9%, F = 7.6%) coffee ≥ 5×/week (M = 44.2%, F = 42.2%)	4809 cognitive healthy subjects Men(M) (*n* = 2077) Women(W) (*n* = 2722)	>65 yrs	FFQ at baseline	Median 7.9 yrs	MMSE	intake of coffee and tea modestly reduced rates of cognitive decline in some women no dose-effect relationship among the women no relationship between caffeine and cognition among men
[42] (Fischer et al., 2018)	Longitudinal epidemiological study	APOE ϵ4 carrier (*n* = 551) APOE ϵ4 non-carrier (*n* = 2071)	2622 dementia-free participants	Avg = 81.2 ± 3.4 yrs Carrier = 80.9 ± 3.4 yrs Non-carrier = 81.3 ± 3.4 yrs	8-item cognitive health food questionnaire at baseline	10 yrs	CERAD SIDAM	no association between coffee consumption and the incidence of dementia/AD
[30] (Santos et al., 2010)	Longitudinal epidemiological study	Men followed up (*n* = 128) Women followed up (*n* = 181)	648 subjects recruited Followed up (*n* = 309) Not followed up (*n* = 339)	≥ 75 yrs. Men = 70(67–73) Women = 71(68–74.5)	FFQ at baseline	2–9 yrs	MMSE	caffeine intake was correlated with reduced cognitive decline among women. no correlation found among men
[43] (Ritchie et al., 2014)	Cross-sectional epidemiological study	Men (*n* = 473) Women (*n* = 720)	1193 cognitive healthy subjects with plasma AB levels	≥65 yrs	Caffeine questionnaire at baseline interview	Nill	*Aβ* levels MMSE	no statistically significant association between caffeine and depression or *Aβ* levels
[44] (Kyle et al., 2010)	Cross-sectional epidemiological study	professional (*n* = 94) skilled manual (*n* = 180) unskilled manual (*n* = 77)	351 subjects born in 1936 and sat the MHT	64 yrs	MONICA food frequency questionnaire	NIll	MMSE	coffee no relationship cognition once account for cognition
[31] (Kim et al., 2019)	Cross-sectional epidemiological study	<2 cups/day (*n* = 269) ≥2 cups/day (*n* = 142)	411 adults without dementia	<2 cups/day = 71.06 ± 7.73 yrs. ≥2 cups/day = 69.67 ± 8.43 yrs	Coffee intake questions in interview	Nill	PET scan MRI scan CERAD-K	higher coffee intake was significantly associated with lower *Aβ* positivity coffee intake was not-associated with hypometabolism, AD-signature region, and WMH volume
[32] (Al-khateeb et al., 2014)	Cross-sectional epidemiological study	Dementia patient (D), (*n* = 52) Healthy Control (C), (*n* = 50)	102 subjects without statins use or substance abuse history	>60 yrs. (C) = 68.9 ± 7.11 yrs. (D) = 70.7± 7.63 yrs	Lifestyle questionnaire at baseline	Nill	MMSE CDT	an incidental finding that increased coffee intake demonstrated a 6.25-fold lower risk for cognitive decline
[33] (West et al., 2019)	Cross-sectional epidemiological study	young group (*n* = 317) older group (*n* = 321)	634 cognitively healthy T2D patients	Young group = 64–71.5 Older group = 71.5–84	FFQ baseline	Nill	episodic memory executive function semantic categorization6 working memory MR imaging	higher caffeine intake was related to better overall cognition effect on cognition was amplified in the older group (above median) compared to the younger
[45] (Cornelis et al., 2020)	Cross-sectional epidemiological study	tea/coffee None/day <1 cup/day 1 cup/day 2–3 cups/day 4–5 cups/day 6–7 cups/day ≥8 cups/day	493,944 subjects without self-reported neurological disease	35–73 yrs	Touchscreen questionnaire	Nill	PM Pairs FI RT SDS	coffee intake significantly decreased reaction time, pairs matching, Trail making test B, and symbol digit substitution No relationship was identified between cognitive function and CMS
[46] (Cornelis et al., 2020)	Cross-sectional epidemiological study	Recent caffeine, whites NO (*n* = 401,650) Recent caffeine, whites YES (*n* = 8533) Recent caffeine, non-whites NO (*n* = 24,152) Recent caffeine, non-whites YES (*n* = 565)	434,900 subjects without self-reported neurological disease	35–73 yrs	Touchscreen questionnaire	Nill	PM Pairs FI RT	Among white and non-white participant, recent coffee consumption was correlated with higher RT performance but worse FI, Pairs, and PM performance
[34] (Iranpour et al., 2020)	Cross-sectional epidemiological study	Q1 caffeine intake Q2 caffeine intake Q3 caffeine intake Q4 caffeine intake	1440 subjects	≥65 yrs Mean = 69.14 yrs	24-hr dietary recall survey	Nill	CERAD DSST	Caffeine intake was significant associated with improved CERAD word recall test this trend was enhanced among men
[35] (Ritchie et al., 2010)	Cross-sectional epidemiological study	Men (*n* = 317) Women (*n* = 324)	641 subjects	≥65 yrs	Caffeine intake questions in interview	Nill	MR imaging	mean log transformed WML/cranial volume ratio was lower for female chronic coffee consumer relationship did not extend to men increased cerebral perfusion in chronic coffee consumers
[47] (Kim et al., 2015)	Cross-sectional epidemiological study	MFDF (*n* = 589) WNC (*n* = 176)	765 cognitive healthy subjects	≥60 yrs	FFQ	Nill	MMSE-KC CERAD	MFDF diet showed a lower risk of cognitive impairment compared to the western diet
[48] (Hosking et al., 2014)	Cross-sectional epidemiological study	Vegetable and non-processed diet ‘Traditional Australian diet,’ ‘non-traditional Australian diet’ ‘Coffee high-fat sugar extras diet Processed, high fat sugar extras diet	352 cognitive healthy subjects	65–90 yrs. Mean 73.12 (SD = 5.47) yrs.	Lifetime diet questionnaire (LDQ)	Nill	MMSE	Coffee, high sugar, high fat diet had worse cognitive performance compared to the ‘vegetable and non-processed diet’
[36] (Dong et al., 2020)	Cross-sectional epidemiological study	0 g/day (*n* = 710) 1 to <266.4 g/day (*n* = 602) 266.4 to <495 (*n* = 607) ≥495 g/day (*n* = 594)	2513 subjects	≥60 yrs	2 × 4 h dietary recall interview	Nill	CERAD DSST Animal fluency test	Caffeinated coffee was associated with improved cognition No association between decaffeinated coffee and cognition
[37] (Lin et al., 2021)	Randomized control study	caffeine treatment (*n* = 10) placebo treatment (n = 10)	20 healthy subjects	18–35 yrs	>3 × 150 mg/day caffeine tablets >Sweat test- caffeine metabolite measurement	5.5 h	MRI ECG	Higher caffeine intake is associated with reduced GMV in the medial temporal lobe Sleep pattern not affected by caffeine treatment
[38] (Haller et al., 2014)	Randomized control study	17 HC 17 MCI	34 subjects without neurological/psychiatric history	HC = 68.3 ± 2.8 yrs MCI = 70.7 ± 4.6 yrs	>Caffeine tablets (200 mg) >placebo tablets	30 min	MMSE CDR HAD LIDA	acute caffeine administration induced a prefrontal activation in HC and a more diffuse posteromedial activation in MCI posterior displacement of working memory-r after caffeine administrations in MCI represents a compensatory mechanism to counterbalance frontal lobe dysfunction

The study did not find a neuroprotective caffeine link 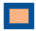; the study found a neuroprotective caffeine link: 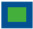.

## 4. In Vitro and In Vivo Studies

Due to unavoidable confounding in clinical studies, animal models have been used to study the relationship between caffeine consumption on AD and cognition while exploring possible action mechanisms. In this review, we identified and included 21 in vivo and in vitro studies that directly explored the effect of caffeine in relation to AD. Twenty of these studies showed caffeine was neuroprotective, and one determined that caffeine has no effect on AD. We used this data to elaborate on the possible mechanism of action of the neuroprotective effect of caffeine. Numerous studies examined other active components in coffee that might be neuroprotective against AD, but these were excluded as this is outside the scope of this review.

### 4.1. Effecting Membrane

Gastaldo et al., investigated the interaction of resveratrol, caffeine, *β*-carotene, and epigallocatechin gallate (EGCG) on peptide aggregate by using synthetic membranes that contained cross-*β* sheets of the membrane active fragment *Aβ*_25–35_ [49]. The effect on the size and volume fraction of *Aβ* fragments was noted using microscopy (optical and fluoroscopy), X-ray diffraction, UV-vis spectroscopy, and molecular dynamic simulations [49]. Gastaldo et al., found that caffeine was membrane-active and simultaneously partitioned into the synthetic membrane, where caffeine caused membrane thickening (38.3 Å ± 0.2) and reduced membrane fluidity but did not affect the volume fraction of peptide aggregates [49]. The study also reported that caffeine attracted water and promoted the expulsion of plaques from the membrane leading to more pronounced amyloid fibrils confirmed by microscopy and x-ray diffraction [49]. The exact role this effect of caffeine plays in neuroprotective AD is unclear, but membranes are reported to play a crucial role in the early stages of peptide aggregation, where they provide a stable base for the crosslinking of neighboring *Aβ* monomers thus by, caffeine causing early expulsion of peptides it prevents crosslinking with neighboring monomers [49].

### 4.2. Altering APP Processing

Janitschke et al., studied the affinity of natural methylxanthines (MTX) (caffeine, theobromine, theophylline) and synthetic MTX (propentofylline, pentoxifylline) on amyloid precursor protein (APP) processing; also, further explored the molecular mechanism of caffeine in the protective effect in AD [50]. They used human neuroblastoma (SH-SY5Y WT and SH-SY5Y APP^695^), which was incubated in MTX (0.1 nmol per 1 μg protein) for 30 min before *α* and *β*-secretase activity measurements using Western blots (WB) [50]. Caffeine increased *α*-secreted APP to (131 ± 9.5%) and decreased direct *β*-secretase activity (93.6 ± 1.0%), decreased BAEC1 gene expression (80.8 ± 6.8%) in SH-SY5Y APP^695^ [50]. Caffeine also increased direct *α*-secretase activity to (112.3 ± 3.0%) and increased ADAM10 protein levels (229.3 ± 22.4%) in SH-SY5Y WT [50]. Indicating that caffeine decreased total secreted *A*β (by 15.5%) levels through elevation of non-amyloidogenic α-secretase APP processing [50]. Furthermore, the study also reported that caffeine reduced reactive oxidative species (ROS) to (48.3 ± 1.6%), reduced cholesterol levels (82.8 ± 3.7%), and reduced *A*β aggregation too (46.7 ± 9.6%) [50]. A major strength of this study is that it explores the molecular mechanism of reduction in *Aβ* levels in the hippocampus reported in caffeine-treated APPswedish transgenic mice [51]. However, as an ex vivo research, the study does not account for BBB and liver metabolism, limiting the results generalizability [50].

Arendash et al., investigated the long-term effect of chronic caffeine administration (1.5 mg/day in drinking water, 4–9 months of age) on transgenic APPswedish mice (Tg) [51]. During the last eight weeks of the study, the mice were subjected to behavioral assessment (open-field task, balance beam task, string-suspension task, Y-maze task, elevated plus-maze, Morris water maze, circular platform task, platform recognition task, radial arm water maze) and the rodents’ post-mortem brains were also analyzed to assess soluble/insoluble *Aβ* levels, PCS1, and BACE levels, adenosine receptor density by WB, and measure of brain adenosine levels [51]. The study also examined the effect of caffeine (0–10 μM) on *A*β production in vivo in APPswedish mice and *A*β generalization in N2a neuronal cultures [51]. The Tg mice with chronic caffeine administration performed significantly better than control Tg mice and, like WT mice, across multiple cognitive domains (spatial learning/reference memory, working memory, recognition/identification) [51]. Post-mortem analysis revealed that caffeine-treated Tg mice had lower hippocampal Aβ-levels, reduced presenilin 1/γ-secretase (PS1) and β-secretase (BACE1) levels, restored brain adenosine levels, and unchanged A_1_ and A_2A_ receptor density compared to control Tg mice [51]. In vitro APPswedish mice and N2a neuronal cultures showed a caffeine concentration-dependent decrease of Aβ production (Aβ_1–40_ and Aβ_1–42_) [51]. This study suggests that the neuroprotective effect of caffeine is likely due to decreased *Aβ* production achieved through modulation of BASE activity [51]. This study was the first to examine the long-term effect of caffeine administration on WT and Tg mice [51]. However, further research is required to explain the mechanisms involved in the modulation of BACE1 and PS1 levels and activity [51].

In another study, Arendash et al., examined the effect of caffeine administration (0.3 mg/mL) on aged transgenic APPswedish mice (18–19 months) showing impaired working memory; after 4–5 weeks of caffeine treatment, the rodents were subjected to behavioral testing (open-field task, balance beam task, string-suspension task, Y-maze task, elevated plus-maze, Morris water maze, circular platform task, platform recognition task, radial arm water maze) and the rodents’ post-mortem brains were also analyzed (immunohistochemistry, *A*β ELISA, pcRaf-1 and PKA analysis) [52]. This study also included a second experiment where 9-month-old Tg mice were gavaged with caffeine (1.5 mg/twice daily for two weeks), after which the mice were sacrificed and subjected to pcRaf-1 and PKA analysis [52]. In the third experiment carried out part of this study, 5.5-month WT mice were put on caffeine (0.3 mg/L) and at the age of 15–16 months were subjected to 6-week behavioral screening [52]. Finally, the study also looked at the effect of concentration-dependent caffeine administration (0–20 μM for 1h) or time-dependent caffeine administration (20 μM for 0–180 min) on APPswedish mice N2a neuronal cultures [52]. Arendash et al., found that caffeine administration on aging Tg mice with impaired cognition showed markedly improved working memory and overall cognition than Tg control mice (*p* < 0.05), who showed continued deterioration [52]. Caffeinated Tg mice had lower *A*β deposition in the hippocampus (−40%) and entorhinal cortex (−46%) and reduced soluble *Aβ* levels than Tg control [52]. Mechanistically they found that BACE1 suppression in Tg caffeinated involves cRaf-1/NF*ηB* pathway (−27%, significantly lower than in TG controls) and PKA (+25%, significantly higher than TG control) and that the physiological concentration of caffeine (1–2 cups) was sufficient to reduce glycogen synthases kinase 3 levels in N2a cells [52]. They also reported no cognitive benefit of long-term caffeine treatment in WT mice [52]. The strength of this study is that it is the first to show that caffeine can reverse AD cognitive impairment and proposes a mechanism of action for the reduction of BAES1 [52].

Cao et al., studied the effect of acute (1.5 mg caffeine IP or gavage) and chronic caffeine administration (2× daily 1.5 mg caffeine gavage for 7 days) on *A*β levels (plasma, CSF, deposition) in transgenic APPswedish mice (Tg) and non-transgenic mice (NT) [53]. The study also carried out in vivo microdialysis of living rodent hippocampus to study the effect of acute caffeine administration on interstitial fluid *A*β levels of the hippocampus by analyzing CSF and collected blood samples [53]. All the plasma samples underwent neurochemical assessments (*A*β levels and cytokine expression profiles), and the chronic caffeine group was also subjected radial arm water maze cognitive test [53]. Cao et al., acute caffeine administration in Tg mice led to reducing *A*β levels in brain interstitial fluid and plasma without affecting *A*β clearance [53]. Chronic caffeine administration led to reduced plasma Aβ, and decreased soluble and deposited Aβ hippocampus and cortex [53]. Plasma *A*β or caffeine levels did not correlate with brain *A*β levels or cognitive performance [53]. However, higher plasma caffeine was linked to reduced hippocampal neuroinflammatory markers [53].

### 4.3. Altering Excitation and Inhibition

A study investigated the long-term effect of early-life exposure to caffeine in THY-Tau22 transgenic mice, an AD Tau pathology model [54]. A caffeine dose of 3 g/L (=humans 4 cup/day) was given to (THY-Tau22 and WT mice) caffeine group, starting 2 weeks before mating to postnatal day 15, after which the offspring were given pure water, and the water group (THY-Tau22 and WT mice) was never exposed to caffeine [54]. The mice’s deficit in learning was accessed at 8 and 12 months using the Barnes maze test, in vitro electrophysiology assessment of hippocampal CA1 pyramidal cells, then the tissue was harvested for further biochemical and molecular evaluation [54]. The THY-Tau22 mice caffeinated offspring developed cognitive deficits (spatial memory and learning) earlier at eight months than water treated offspring at 12 months [54]. WT mice showed no difference between caffeinated and water groups, unlike in Silva et al., but this is thought to be because object location memory was not tested in this study [54,74]. There was no correlation between cardinal PTau, neuroinflammatory markers, and memory deficit in caffeinated offspring [54]. In vitro electrophysiology assessment showed that early life caffeine exposure altered how glutamatergic and GABAergic circuits were affected by Tau pathology [54]. At eight months, caffeinated Tau mice had lower glutamatergic and GABAergic neuron function, while at 12 months, their excitatory drives were decreased. Still, inhibitory drives were increased compared to water Tau mice which had higher glutamatergic and GABAergic drives compared to water WT mice at 8 and 12 months [54]. Thus, indicating a more complex non-linear Tau-age-caffeine interaction than the predicted simple caffeine-induced aging-like increase in glutamatergic and GABAergic drives of Tau mice [54]. The limitation of this study is that only 2-time points of electrophysiology assessment were conducted, which is insufficient to describe caffeine-induced changes in glutamatergic and GABAergic drives of Tau mice; more in vivo electrophysical measurements are warranted to study this hypothesis further [54].

### 4.4. Altering Protein Aggregation

Mancini et al., examined the ability of six compounds in coffee (caffeine, chlorogenic acid, quinic acid, caffeic acid, quercetin, and phenylindole) at 25 mM to inhibit fibrilization of *A*β and Tau or a-synuclein using thioflavin T (ThT) and thioflavin S (ThS) fluorescence assay [55]. All instant coffee (light, dark and roast) inhibited Aβ and Tau aggregation at 100 μg/mL [55]. However, caffeine had no effect on Aβ, Tau, and a-synuclein with no measurable IC_50_ values [55]. This study’s limitation is that higher concertation of caffeine that might inhibit aggregation was not tested [55]. However, other coffee compounds are more potent inhibitors of protein aggregation, such as phenylindanes (inhibited both Aβ and Tau fibrilization and Aβ oligomerization), and decaffeinated and caffeinated coffee exhibit similar levels of inhibition of protein aggregation [55].

Laurent et al., investigated the effect of chronic caffeine intake (0.3 g/L drinking water) on THY-Tau22 transgenic mouse’s progression of Tau pathology [56]. The rodents were subjected to a Morris water maze cognitive test, biochemical analysis, mRNA extraction, and caffeine metabolite sampling (brain and plasma) [56]. Chronic caffeine-exposed (32.30 ± 2.21%) mice performed significantly better than control transgenic mice (25.56 ± 2.57%) in spatial memory tests and were comparable to WT water administered mice (34.04 ± 3.37%) [56]. Furthermore, the study reported that caffeine administration to WT mice did not improve its cognitive performance [56]. Caffeinated THY-Tau22 mice (−22.6 ± 7.0%) also had significantly lower Tau phosphor-isotopes than THY-Tau22 control mice [56]. Caffeinated THY-Tau22 mice also had significantly lower pro-inflammatory protein and oxidative stress markers than control Tau mice [56]. Importantly, this is the first study to demonstrate the neuroprotective effect of caffeine against Tau pathology [56].

### 4.5. Antioxidant Properties

Alzoubi et al., looked at the ability of caffeine (0.3 g/mL added to drinking water) to reduce the cognitive decline caused by increased oxidative stress due to administration of L-methionine (1.7 g/kg/day orally) for a treatment period of 4 weeks [57]. Then radial arm water maze (RAWM) was used to measure cognition (spatial learning and memory), and a calorimetric immunoassay was used to measure hippocampal tissue antioxidant biomarker [57]. Alzoubi et al., reported that L-methionine administration caused (short and long) term memory impairment (*p* < 0.05) while caffeine negated that effect [57]. L-methionine administration caused reduced catalase and glutathione peroxidase (GPx) enzyme activities; reduced glutathione (GSH) oxidized glutathione (GSSG) ratio compared to controls, while caffeine administration normalized these effects [57]. A strength of this study is that it directly examined the effect of caffeine on the hippocampus antioxidative protection system (GSH/GSSG ratio, catalyze, GPx), allowing for a causal relationship to be determined [57].

### 4.6. Effect on BNDF Levels

Additionally, two studies also showed through in vivo experiments that caffeine was able to prevent or reversed the reduction in brain-derived neurotrophic factor (BNDF) in AD mice and mice on a high-fat diet (Table 2) [58,59]. This could be a key mechanism in understanding the neuroprotective effect of caffeine in AD.

### 4.7. AR Antagonist Properties

Zhao et al., investigated if administration of 3 g/L caffeine (non-selective antagonist of A_2A_R) in drinking water or gene knockout (A_2A_R KO mouse model) can elevate cognitive impairment by reducing Tau-hyperphosphorylation induced by traumatic brain injury (TBI) using a mouse model of moderate cortical impact [60]. The mice’s cognition was assessed using the Morris water maze test (day 7 and week 4), and post-mortem (immunofluorescence, immunohistochemistry, Golgi staining, western blot) analysis was also performed [60]. TBI-induced PTau was confirmed by increased Ser404 close to the site of injury in the dentate gyrus of the contralateral hippocampus (24 h, 7 days, 4 weeks post-TBI) and spatial memory impairment in Morris test (7 days and 4 weeks) [60]. Chronic Caffeine treatment (starting 3-week before TBI) prevented TBI-induced PTau and spatial memory deficit (7 days and 4 weeks) [60]. The study proposes a novel post-TBI (TBI-common among AD patients 20–30%) mechanism of A_2A_R activation that triggers Tau hyperphosphorylation, causing memory impairment which may be normalized by chronic caffeine administration [60]. Further research is required to explore the underlying mechanism for the protective effect of A_2A_R on certain types of memory and why hyperphosphorylated Tau appears in specific brain regions [60].

Bortolotto et al., investigated the effects of acute of caffeine (10 mg/kg, non-selective AR antagonist), ZM241385 (10 μg/kg, A_2A_R antagonist), DPCPX (0.5 mg/kg, A_1A_R antagonist), dipyridamole (5 mg/kg, nucleoside transporter inhibitor), ELINA (100 μg/kg, adenosine deaminase) on scopolamine (200 μM) induced memory loss in adult WT Zebrafish [61]. Then the fish was subjected to a battery of behavioral tests: inhibitory avoidance task, exploratory assessment, and social interaction test [61]. Interestingly, caffeine pre-treatment prevented scopolamine-induced amnesia (*p* < 0.0001 diff between training and test) in the inhibitory avoidance test [61]. Caffeine administration also did not cause a significant change in social interaction or exploratory assessment [61]. Other substances tested also showed neuroprotective effects against scopolamine-induced memory deficits showing that adenosine blockage can stop scopolamine-induced amnesia [61]. Caffeine’s ability to prevent scopolamine-induced amnesia has been previously explored in rodent studies [61].

Li et al., studied if caffeine, through A_3_R action, can reduce *A*β precursor protein (AβPP) and LDL internalization, therefore reducing *A*β generation [62]. This study used rat embryonic primary cerebral cortical neurons and human blastoma SH-SY5Y (expressing WT *A*β). [62]. All the cells were then treated with LDL (1 μg/mL) to measure LDL internalization (known AD risk factor) [62]. Then the cells were selectively exposed to the following treatment: caffeine (200μM), selective AR-1a,2a,2b,3r antagonists, A_3_R gene knockout treatment. Then the cells were observed: immunoblotting, surface immunostaining, RT-PCR, Lactate dehydrogenase (LDII- cell injury marker) [62]. Li et al., reported that caffeine, A_3_R antagonist, A_3_R gene knockout showed a concentration-dependent reduction in LDL internalization, suppression of LDL induced Aβ generation, and suppressed *A*βPP internalization [62]. Interestingly caffeine suppressed LDL-induced *A*β_1–40 & 1–42,_ but A_3_R gene knockout only suppressed *A*β_1–40_ [62]. Therefore, this study proposes a novel mechanism where caffeine protects against LDL-enhanced *A*βPP internalization and processing into *A*β by A_3_R antagonism [62].

Espinosa et al., studied the effect of caffeine consumption (30 μM plasma) in adult Wistar rats with sporadic AD (induced by streptozotocin (STZ), 5 μL) and its effect on memory impairment, neuronal damage, A_2A_R hippocampal density [63]. After treatments, the rodents were subjected to cognitive tests (behavioral analysis, novel object recognition task), immunohistochemistry, immunoblotting, and quantitative-PCR [63]. Espinosa et al., reported that caffeine treatment prevented memory decline induced by STZ (F_(1,38)_ = 46.195, *p* < 0.001) but had no effect on controls [63]. Caffeine treatment was also shown to prevent neurodegeneration induced by STZ in NeuN immunohistochemistry (F_(1,8)_ = 12.49, *p* < 0.0077), WBs also showed that caffeine prevented STZ induced increased A_2A_R hippocampal density (F_(1,20)_ = 4.83, *p* < 0.0399) but had no effect on controls [63]. This study demonstrates that caffeine can prevent STZ-induced memory decline while simultaneously controlling the hippocampal A_2A_R population, but the underlying mechanism for this neuroprotective effect was not explored [63].

Dall’lgna et al., used CF1 adult mice with cognitive decline induced by Aβ injection (3 nmol) and treated them with caffeine: acute, subchronic, prolonged, or combined [64]. Prolonged treatment consisted of (1 mg/mL of caffeine in drinking water for 12 days before *A*β administration on day 7), sub-chronic caffeine administration (30 mg/kg caffeine for 2 days before and one day after Aβ administration), acute caffeine treatment intraperitoneal (80 mg/kg caffeine, 30 min before *A*β administration), combined prolonged and acute (1 mg/mL of caffeine in drinking water for 12 days and 80 mg/kg caffeine, 30 min before Aβ administration) [64]. The same protocol of subchronic caffeine administration was followed for selective A_2A_R antagonist (SCH58261, 0.5 mg/kg dose) [64]. Aβ administration caused impaired performance in inhibitory avoidance and spontaneous alteration [64]. Acute caffeine administration alone did not stop the Aβ-induced impaired performance, but prolonged caffeine or selective A_2A_R treatment using (subchronic, chronic, and combined prolonged) protocols showed a protective effect against cognitive decline [64].

### 4.8. Effect on Endolysosomes Dysfunction

Soliman et al., treated SH-SY5Y (over-expresses *A*β*PP*) with HIV-1 transactivator of transcription (Tat) (200 μM) for two days in the presence/absence of caffeine (200 μM) [65]. Then they used quantified *A*β levels, vacuolar-ATPase protein, and phosphorylated Tau levels [65]. Soliman et al., reported that HIV-1 Tat significantly increased levels of secreted and intracellular levels of *A*β as well as hyperphosphorylated Tau [65]. HIV-1 Tat also significantly reduced levels of vacuolar-ATPase, a major pathway that Endolysosomes use to maintain an acidic environment [65]. Treatment with caffeine prevented this HIV-1 Tat-induced increase in *A*β and Tau levels and prevented a decrease in vacuolar-ATPase [65]. The study hypothesized that caffeine could reduce HIV-1 Tat-induced lysosomal dysfunction, which allows the Endolysosomes to secrete H+ and maintain a lower PH. This helps prevent an increase in *A*β and Tau levels [65]. Important to note that this study used 200 μM of caffeine in the upper range of free caffeine plasma concentration, far higher than the 1–10 μM free caffeine plasma concentration seen after one cup of coffee [65].

### 4.9. Acetylcholinesterase Inhibition

Additionally, two studies showed that caffeine could inhibit acetylcholinesterase (AChE) through in vitro experiments and computer modeling, which might be an important pathway for the neuroprotective effect of caffeine. However, these studies also reported the plasma caffeine concentration needed for this effect is higher than normal plasma concentration after consumption of coffee or energy drink [66,67].

### 4.10. Effect on Granulocyte-Colony Stimulating Factor, IL-6, and IL-10

Cao et al., examined the acute effects of decaffeinated coffee, caffeinated coffee (1.5 mg caffeine), and pure caffeine (1.5 mg caffeine) on plasma cytokines measured (pre-treatment and 30 min post-treatment) of 8-month-old transgenic APPswedish mice (Tg) and 8-month-old non-transgenic mice (NTg) [68]. This study also examined the chronic effects of decaffeinated coffee, caffeinated coffee (0.75 mg caffeine), saline, and pure caffeine (0.75 mg caffeine) administered twice weekly through gavage on plasma cytokines measured (on the 13th month) of 10-month-old Tg and 10-month-old NTg [68]. Rodents were subjected to cognitive interference task (chronic study only), and blood samples were analyzed using Luminex assay (12 cytokines and chemokines), ELISA (plasma cons of theophylline, caffeine, and *A*β levels) [68]. The study reported that in the acute treatment, plasma levels of a granulocyte-colony stimulating factor (GCSF), IL-6, and IL-10 were elevated for Tg and NT mice treated with caffeinated coffee, not for caffeine treatment or decaffeinated coffee treatment [68]. The chronic experiment identified that both caffeinated coffee and caffeine treatment allowed better working memory preservation than Tg controls and to the same level as NT controls [68]. This experiment also identified higher plasma GCSF levels correlated with better cognition [68]. The mechanism of GCSF neuroprotection is hypothesized to be through the recruitment of microglia from bone marrow, synaptogenesis, and neurogenesis [68]. The strength of this study is that it demonstrated the importance of the source of caffeine and how caffeine from coffee might have additional benefits due to synergetic effects with an unknown component [68].

### 4.11. Effect on Aβ–Clearance

Qosa et al., investigated the potential of caffeine and rifampicin to enhance Aβ clearance (induced by 30 nM of ^125^I-*Aβ*_40_) across BBB in C57BL/6 WT mice [69]. For the in vivo experiment, they treated WT mice with caffeine/rifampicin (20 mg/kg intraperitoneally, 2-week caffeine, and 3-week rifampicin) and subjected them to a brain efflux index study [69]. Then for the In vitro experiment, they treated mouse brain endothelial cells (bEnd3) with 50 μM of caffeine/rifampicin and analyzed them using PT-PCR and western blotting [69]. Qosa et al., reported the brains of mice treated with caffeine (BEI% = 80.4 ± 4.3%, p<0.01) showed significantly higher *Aβ* clearance across BBB compared to controls (BEI% = 62.4 ± 6.1%, p<0.01) [69]. The in vitro studies also demonstrated that caffeine treatment significantly upregulates the expression of P-glycoprotein (P-GP), which is thought to be part of the mechanism that increases *A*β across BBB [69]. The importance of this study is that it clearly demonstrates that the beneficial effects of caffeine can be extended to clearance across BBB and identified the presence of another unidentified transport/receptor protein that acts in the same direction of low-density lipoprotein receptor (LRPI) [69].

### 4.12. Studies Reporting no Effect of Caffeine

Shukitt-Hale et al., examined the effect of caffeine and coffee on cognition in WT Fisher mice [70]. The mice were subjected for 8 weeks to varying diets of coffee (0, 0.165, 0.275, 0.55, 0.825) % of coffee extract in study 1 and for study 2 they used (0.387, 0.55)% coffee and (0.0181, 0.0258)% caffeine [70]. The rodents were then subjected to a battery of psychomotor (rod walking, wire suspension, plank walking, inclined screen, accelerating rod), cognitive (Morris water maze), caffeine, and hydroxycinnamic acid concentration sampling [70]. The study reported that 0.55% and 0.165% of coffee proved optimal for working and reference memory [70]. However, in study 2, which compared the effect of pure caffeine, it was found that it did not fully account for the protective effect of coffee, indicating that there might be other players to the neuroprotective effect of coffee [70].

**Table 2:** In vitro and in vivo studies summary.

Study	In-Vivo/In Vitro Study	Mechanism of Neuroprotective Effect	Study Methodology	Main Outcomes
[49] Gastaldo et al., 2020	In Vitro	Effecting membrane	➢ Studied interaction of resveratrol, caffeine, carotene, and epigallocatechin gallate (EGCG) on *Aβ* peptide aggregate by using synthetic membranes that contained cross-sheets of *Aβ* 25–35 ➢ The effect on the size and volume fraction of *Aβ* fragments noted using microscopy, x-ray diffraction, UV-vis spectroscopy, and molecular dynamic simulations	➢ caffeine was membrane-active and simultaneously partitioned into the synthetic membrane, where caffeine caused membrane thickening ➢ caffeine attracted water and promoted the expulsion of plaques from the membrane leading to more pronounced amyloid fibrils ➢ caffeine by causing early expulsion of peptides prevents crosslinking with neighboring monomers and reduces peptide aggregation
[50] Janitschke et al., 2019	In Vitro	Altering APP processing	➢ human neuroblastoma (SH-SY5Y WT and SH-SY5Y APP695) was incubated in MTX (0.1 nmol per 1 g protein) 30 min before α and β-secretase activity measurements using WB	➢ caffeine decreased total secreted *Aβ* levels by 15.5% through elevation of non-amyloidogenic α-secretase APP processing ➢ caffeine reduced ROS, cholesterol levels, and *Aβ* aggregation
[51] Arendash et al., 2006	In Vivo	Altering APP processing	➢ studied the effect of chronic caffeine administration (1.5 mg/day in drinking water, 4–9 months of age) on transgenic APPswedish mice (Tg) ➢ last 8 weeks of the study, the mice were subjected to behavioral assessment ➢ rodent’s brain subjected to post-mortem WB analysis to measure soluble/insoluble *Aβ* levels, PCS1, BACE and adenosine levels, and adenosine receptor density	➢ Tg mice with chronic caffeine administration performed significantly better than control Tg mice across multiple cognitive domains ➢ caffeine treated Tg mice had lower hippocampal *Aβ* levels, reduced presenilin 1 (PS1) and β-secretase (BACE1) levels, restored brain adenosine levels, and unchanged A1 and A2A receptor density compared to control Tg mice
	In Vitro		➢ Studied effect of caffeine (0–10 μM) on *Aβ* production in vivo in APPswedish mice	➢ APPswedish mice and N2a neuronal cultures showed a caffeine concentration-dependent decrease in *Aβ*1–40 and *Aβ*1–42 production
[52] Arendash et al., 2009	In Vivo	Altering APP processing	➢ effect of caffeine administration (0.3 mg/mL) on aged transgenic APPswedish mice (18–19 months) showing impaired working memory ➢ after 4–5 weeks of caffeine treatment, the mice were subjected to behavioral testing ➢ post-mortem tissue was subjected to immunohistochemistry, *Aβ* ELISA, pcRaf-1, and PKA analysis	➢ caffeine administration on aging Tg mice showed markedly improved working memory and overall cognition than Tg control mice (*p* < 0.05) ➢ caffeinated Tg mice had lower *Aβ* deposition and lowered soluble *Aβ* levels than Tg control ➢ mechanistically the neuroprotective effect of caffeine involves BACE1 suppression in Tg caffeinated through cRaf-1/NFηB pathway and PKA
			➢ 9-month-old Tg mice were gavage with caffeine (1.5 mg/twice daily for 2 weeks), ➢ sacrificed and subjected to pcRaf-1 and PKA analysis	
			➢ 5.5-month WT mice were put on caffeine (0.3 mg/L) ➢ age of 15–16 months was subjected to 6-week behavioral screening	
	In Vitro		➢ effect of concentration-dependent caffeine administration (0–20 μM for 1h) or time-dependent caffeine administration (20 μM for 0–180 min) on APPswedish mice N2a neuronal cultures	➢ there was reduce glycogen synthases kinase 3 levels in N2a cells
[53] Cao et al., 2009	In Vivo	Altering APP processing	➢ acute (1.5 mg caffeine IP or gavage) and chronic caffeine administration (2× daily 1.5 mg caffeine gavage for 7 days) on *Aβ* levels of (Tg) and (NT)	➢ acute and chronic caffeine administration in Tg mice led to reduced *Aβ* levels in brain interstitial fluid and plasma
			➢ microdialysis of living rodent hippocampus to study the effect of acute caffeine administration on interstitial fluid *Aβ* levels	➢ plasma *Aβ* or caffeine levels did not correlate with brain *Aβ* levels or cognitive performance
[54] Zappettini et al., 2019	In Vivo	Altering excitation and inhibition	➢ investigated the long-term effect of early-life exposure to caffeine in THY-Tau22 transgenic mice ➢ caffeine dose of 3 g/L was given to parental THY-Tau22 Mice and WT mice, starting 2 weeks before mating and continued to postnatal day 15 ➢ then learning of offspring Tg and WT mice was accessed at 8 and 12 months ➢ offspring Tg and WT mice were subjected to in vivo electrophysiology examination of hippocampal neuronal activity and post-mortem biochemical analysis	➢ in vitro electrophysiology assessment showed that early life caffeine exposure altered glutamatergic and GABAergic circuits ➢ complex non-linear Tau-age-caffeine interaction rather than the predicted simple caffeine-induced aging-like increase in glutamatergic and GABAergic drives
[55] Mancini et al., 2018	In Vitro	Altering protein aggregation	➢ studied ability of caffeine, chlorogenic acid, quinic acid, caffeic acid, quercetin, and phenylindole at 25 mM to inhibit fibrilization of *Aβ* and Tau ➢ using (ThT) and (ThS) fluorescence assay	➢ caffeine on its own could not interfere with *Aβ*, Tau aggregation, and *Aβ* oligomerization
[56] Laurent et al., 2014	In Vivo	Altering protein aggregation	➢ effect of chronic caffeine intake (0.3 g/L drinking water) on THY-Tau22 mouse ➢ rodent subjected to cognitive test, biochemical analysis, mRNA extraction, and caffeine metabolite sampling	➢ chronic caffeine Tg mice performed significantly better than control Tg mice ➢ Caffeinated Tg mice had significantly lower Tau phosphor-isotopes, pro-inflammatory and oxidative stress markers than Tg control mice
[57] Alzoubi et al., 2018	In Vivo	Antioxidant properties	➢ caffeine (0.3 g/mL added to drinking water) to reduce the cognitive decline caused by increased oxidative stress due to administration of L-methionine (1.7 g/kg/day orally) for a treatment period of 4 weeks ➢ cognition and hippocampal tissue antioxidant markers were assessed	➢ L-methionine administration caused (short and long) term memory impairment while caffeine negated that effect ➢ L-methionine administration caused reduced catalyze and GPx enzyme activities; reduced GSH, GSSG ratio compared to controls, while caffeine administration normalized these effects
[58] Moy et al., 2013	In Vivo	Effect on BNDF levels	➢ effect of caffeine on rats placed on a high-fat diet ➢ Rodents’ hippocampus was subjected to microdialysis and then spontaneous alternating testing to test the working memory of rodents. ➢ Post mortem the rodent brains were subjected to histology, WB, and enzyme-linked immunosorbent assay (BNDF quantification)	➢ caffeine treatment was sufficient to prevent high-fat diet weight gain and high-fat diet memory impairment ➢ caffeine diet prevented reduction in BNDF induced by a high-fat diet and allowed maintenance of synaptic plasticity
[59] Han et al., 2013	In Vivo	Effect on BNDF levels	➢ effect of caffeine 0.75 mg/day or 1.5 mg/day on saline vehicle treatment for 8 weeks on the expression of BNDF and TrKB receptors in Tg mice ➢ rodents subjected to a Morris water maze test on and WB	➢ caffeine administration Tg mice significantly improved cognitive performance compared to control Tg mice ➢ dose-response increase of hippocampal BNDF and TrKB expression in caffeinated Tg mice
[60] Zhao et al., 2017	In Vivo	AR antagonist properties	➢ administration of 3 g/L caffeine in drinking water or A_2A_R KO mouse model can increase cognitive impairment by reducing Tau-hyperphosphorylation induced by TBI mouse model ➢ cognition was assessed using the Morris water maze test (day 7 and week 4 post-treatment) ➢ post-mortem (immunohistochemistry, Golgi staining, Western blotting were also performed	➢ post-TBI mechanism of A_2A_R activation that triggers hyperphosphorylation of Tau, causing memory impairment may be normalized by chronic caffeine administration
[61] Bortolotto et al., 2015	In Vivo	AR antagonist properties	➢ effects of acute of caffeine (10 mg/kg), ZM241385 (10 μg/kg,), DPCPX (0.5 mg/kg), dipyridamole (5 mg/kg), ELINA (100 μg/kg,) on scopolamine (200 μM) induced memory loss in adult WT Zebrafish ➢ subjected to behavioral tests such as inhibitory avoidance task, exploratory assessment, and social interaction test	➢ caffeine pre-treatment prevented scopolamine-induced amnesia
[62] Li et al., 2015	In Vitro	AR antagonist properties	➢ caffeine (200 μM), selective AR-1a,2a,2b,3r antagonists, A_3_R gene knockout treatment can reduce *AβPP* and LDL internalization, therefore reducing *Aβ* generation in rat embryonic primary cerebral cortical neurons and human blastoma SH-SY5Y ➢ the cells were examined by Western blotting, surface immunostaining, RT-PCR, Lactate dehydrogenase	➢ caffeine, A_3_R antagonist, A_3_R gene knockout showed a concentration-dependent reduction in LDL internalization, suppression of LDL-induced *Aβ* generation, suppressed *AβPP* internalization
[63] Espinosa et al., 2013	In Vivo	AR antagonist properties	➢ effect of caffeine consumption (30 μm plasma) in adult Wistar rats with sporadic AD (induced by STZ, 5 μL) ➢ rodents were subjected to cognitive tests, immunohistochemistry, immunoblotting, and quantitative-PCR	➢ caffeine can prevent STZ-induced memory decline while simultaneously controlling the hippocampal A_2A_R population
[64] Dall’Igna et al., 2007	In-Vivo	AR antagonist properties	➢ CF1 adult mice with cognitive decline induced by *Aβ* injection (3 nmol)and treated them with caffeine or selective A_2A_R treatment (acute, subchronic, prolonged, or combined) ➢ Subjected to inhibitory avoidance and spontaneous alteration cognitive tests	➢ prolonged caffeine or selective A_2A_R treatment using: (subchronic, chronic, and combined prolonged) protocols showed a protective effect against cognitive decline
[65] Soliman et al., 2017	In Vitro	Effect on endolysosomes dysfunction	➢ SH-SY5Y with HIV-1 Tat (200 μM) for 2 days in the presence/absence of caffeine (200 μM) ➢ quantified *Aβ* levels, vacuolar-ATPase, and phosphorylated Tau protein levels	➢ caffeine was able to prevent HIV-1 Tat induced increase in *Aβ* and Tau levels and prevent a decrease in vacuolar-ATPase
[66] Mohamed et al., 2013	In Vitro In Silico	Acetylcholinesterase inhibition	➢ effect of xanthine (caffeine, pentoxifylline, propentofylline) on the inhibition of AChE through in vitro and molecular modeling studies	➢ caffeine was a weak AChE inhibitor
[67] Pohanka et al., 2013	In vitro In silico	Acetylcholinesterase inhibition	➢ used standard Elman test and in silico examinations to determine whether caffeine could inhibit human BChE and ACh	➢ caffeine is a strong non-competitive inhibitor of AChE and a weak non-competitive inhibitor of BChE
[68] Cao et al., 2011	In Vivo	Effect on granulocyte-colony stimulating factor, IL-6, and IL-10	➢ examined the effects of decaffeinated coffee, caffeinated coffee (1.5 mg caffeine), and pure caffeine (1.5 mg caffeine) on plasma cytokines measured in 8-month-old Tg and NTg mice ➢ chronic effects of decaffeinated coffee, caffeinated coffee (0.75 mg caffeine), saline, and pure caffeine (0.75 mg caffeine) administered twice weekly through gavage on plasma cytokines of 10-month-old Tg and NTg ➢ Rodents were subjected to cognitive interference task, and blood samples were analyzed using Luminex assay and ELISA	➢ acute treatment, plasma levels of GCSF, IL-6, IL-10 was elevated for Tg and NTg mice treated with caffeinated coffee only ➢ chronic experiment, it was identified that both caffeinated coffee and caffeine treatment allowed better preservation of working memory compared to NTg controls and higher plasma GCSF levels correlated with better cognition
[69] Qosa et al., 2012	In Vivo In Vitro	Effect on *Aβ*-clearance	➢ treated WT mice with caffeine/rifampicin (20 mg/kg intraperitoneally, 2-week caffeine, and 3-week rifampicin) and subjected them to a brain efflux index study	➢ brains of mice treated with caffeine showed significantly higher *Aβ* clearance across BBB compared to controls
			➢ treated mouse bEnd3 with 50 μM of caffeine/rifampicin and analyzed them using PT-PCR and WB	➢ in vitro caffeine treatment significantly upregulates the expression of P-GP-mechanism that increases *Aβ* across BBB
[70] Shukitt-hale et al., 2013	In Vivo	Nill	➢ WT mice were subjected for 8 weeks to varying diets of coffee extract (0%, 0.165%, 0.275%, 0.55%, 0.825%) in study 1 ➢ study 2 WT mice were subjected to coffee (0.387%, 0.55%) and (0.0181%, 0.0258%) for 8 weeks ➢ then the rodents were subjected to psychomotor and cognitive testing ➢ the brain and serum concentrations of caffeine and hydroxycinnamic acid metabolites were also recorded	➢ pure caffeine did not fully account for the protective effect of coffee

Study did not find a neuroprotective caffeine link: 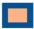; study found a neuroprotective caffeine link: 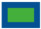.

## 5. Discussion

When considering the results of these studies, variances in caffeine metabolism between individuals that lead to varying plasma caffeine concentration must be considered as it can cause variation in physiological effects. 99% of the caffeine from beverages, i.e., tea and coffee, is rapidly absorbed through the gastrointestinal tract and distributed through body water, bypassing the liver (V = 0.7 L/kg), reaching peak plasma concentration within 15–120 min [75,76]. The main route of caffeine metabolism for clearance in humans (70–80%) is through the N-3-demethylation to paraxanthine pathway, which CYP1A2 carries out in the liver [77]. The existence of multiple variants of CYP1A2 causes variability in caffeine metabolism and plasma caffeine concentration in individuals [78]. Furthermore, smokers’ caffeine metabolism is also accelerated, leading to lower plasma caffeine concentration [79]. Other than that, exogenous estrogen has also been shown to inhibit caffeine metabolism and increase plasma caffeine concentration [79].

This review identified a few studies that examined the neuroprotective effect of coffee/caffeine by using brain imaging (MRI, fMRI) to assess the changes to brain structure and neural activation patterns [25,26,31,35,38,43]. Studies by Haller et al. [25,38] showed in early cognitive decline, there is an increase in compensatory basal activity diffused through the posto-temporal region of the brain, which increases the brain’s sensitivity to the neuroprotective action of caffeine [25,38]. Furthermore, MRI studies by Ritchie et al. [35] and Haller et al. [26] showed that caffeine reduces the amount of white matter lesion/cranial volume in cognitively stable elders, contributing to cognitive decline in Dementia/AD [26,43]. Ritchie et al. [43] also showed increased cerebral perfusion in chronic coffee consumers, indicating a possible neuroprotective mechanism of coffee [43]. Moreover, Gelber et al. [39] found high caffeine levels were associated with a lower-odds of having any brain lesion types at autopsy [39]. However, an epidemiological study by Kim et al. [31] did not find any association between coffee intake and hypometabolism, atrophy of AD signature, and WMH volume; instead, it found that coffee exerted a neuroprotective effect by reducing the levels of Aβ [31].

Current literature also seems to support the notion that caffeine/coffee acts as a cognitive normalizer instead of a cognitive enhancer, and as such healthy adults or those with deteriorating cognition receive little benefit from coffee/caffeine treatment [25,26,33,38]. West et al., found that elderly participants with mild cognitive decline showed higher sensitivity to caffeine than healthy younger diabetics [33]. Furthermore, Haller et al., found no changes in neural activation among healthy adults but increased posto-temporal activation in those with MCI, further supporting the notion that caffeine does not act as a cognitive enhancer [38]. Other than that, caffeine/coffee cannot significantly enhance the cognitive function of those suffering from severe cognitive decline [25,26]. Haller et al., showed that caffeine reduces the amount of white matter lesion/cranial volume and increases cerebral perfusion in cognitively stable elders but did not extend the same benefits to elders with deteriorating cognition [26]. Furthermore, Haler et al., in another study used fMRI to study the neural activation induced by caffeine for participants with deteriorating cognition; although this study showed that caffeine reduced cognitive decline in dCON, it did not show the same level of caffeine-induced neural activation in them as seen in those with sCON [25].

The literature also shows that the neuroprotective effect of caffeine/coffee can be confounded by gender, but the evidence is not definitive for either gender, and further research is needed [24,28,34,43]. Two studies (Ritchie et al. [28,43]) only found a statistically significant neuroprotective effect of caffeine among women in the study population but not males [28,43]. Furthermore, Sugiyama et al. [24] found an overall neuroprotective effect of coffee, but this was enhanced among the female cohort and non-smokers and non-drinkers [24]. However, Iranpour et al., in a crude model, found the neuroprotective effect of caffeine extended to both genders, but after adjusting for confounding, found a weak positive correlation for the neuroprotective effect of caffeine only among the males only [34]. The exact mechanism for the difference in neuroprotective properties of caffeine/coffee between gender is unclear and warrants further research; however, it has been hypothesized that this may be due to differences in caffeine metabolism, pharmacodynamics, or hormonal influence [28,34].

**Figure 1:**
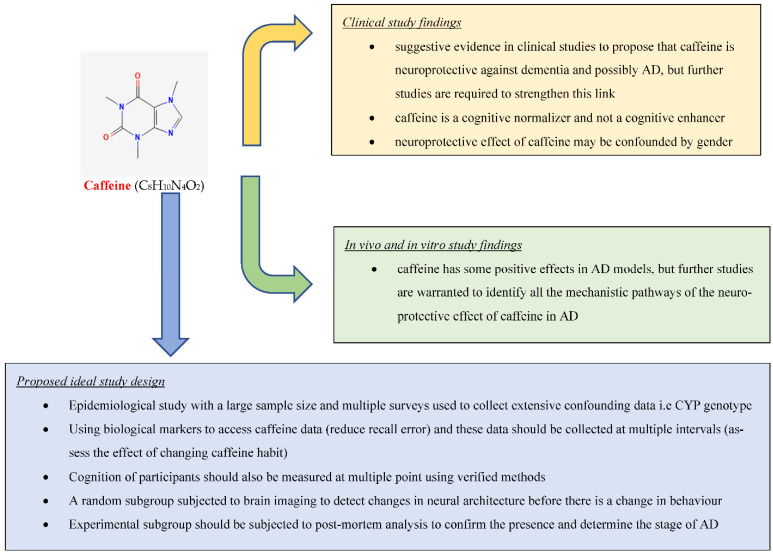
Summary of findings.

Although there is evidence from the clinical studies suggesting that caffeine consumption is protective against AD cognitive decline, further clinical studies are required to prove this link. Ideally, to examine this link, there would need to be an epidemiological study with large sample size, with multiple surveys collecting extensive data on confounding variables to be adjusted, including data on CYP genotype. The study should also use biological markers (blood tests) to assess caffeine to reduce recall bias, and caffeine data should be collected at multiple points (incl: midlife) during extended follow-up to assess changes in behavior. Furthermore, cognition should also be measured using verified methods, i.e., MMSE, CDR, CERAD, during follow-up. A sub-group should also be randomly chosen and subjected to brain imaging at set intervals during follow-up to identify changes in neural architecture before shifting in behavior. This subgroup should also be subjected to post-mortem analysis to confirm the presence and stage of AD (Figure 1).

This review also analyzed in vivo and in vitro studies that directly examined the relationship between caffeine on AD and cognition, which shows strong evidence that caffeine is neuroprotective against AD through multiple mechanisms. These studies also suggest possible mechanisms of caffeine’s neuroprotective effect. Four of these studies show that caffeine alters APP processing to a non-amyloid pathway, reducing AD burden and cognitive decline [50,51,52,53]. Furthermore, five of these studies show that caffeine‘s neuroprotective effect is due to its ability as a non-selective adenosine receptor antagonist [60,61,62,63,64]. Other than that, studies have also shown that caffeine’s neuroprotective effect is due to its ability to alter protein aggregation [55,56]. We also found evidence that caffeine is able to reduce BNDF levels [58,59]. Caffeine also reduces acetylcholinesterase activity, a mechanism for its neuroprotective effect against AD [66,67]. Some studies showed that the neuroprotective effect of caffeine is by: affecting membrane properties [49], changing GABAergic and glutamatergic neurotransmission [54], reducing endolysosome dysfunction [65], increasing GCSF function [68], and increasing *Aβ* clearance [69].

Through this review, we also identified a few in vivo and in vitro studies that were excluded because they did not directly examine the relationship between caffeine and AD. These, however, also posited possible mechanisms of action for the neuroprotective effect of caffeine. Reznikov et al., showed that caffeine significantly enhanced C-Fos expression in the horizontal limb of the diagonal band of Broca [80]. This is thought to explain why AD patients lose their olfactory sense first and that the cognitive enhancing effect of caffeine may be through activation of the basal cholinergic forebrain [80]. Furthermore, Vila-luna et al., showed that the caffeinated group of mice had greater fourth and fifth-order basal dendrites branching in CA1 pyramidal neurons. Laurent et al. showed caffeine’s ability through A2R antagonism/knock out for normalization of hippocampal GSH/GSSG ratio, global reduction in Tau hyperphosphorylation, and neuroinflammatory markers [81].

## 6. Conclusions

This review found suggestive evidence in clinical studies to propose that caffeine is neuroprotective against dementia and possibly AD, but further studies are required to strengthen this link (Figure 1). The clinical studies also point out that caffeine is a cognitive normalizer and not a cognitive enhancer. Although clinical studies show that the neuroprotective effect of caffeine may be confounded by gender, it is not conclusive. The review also found strong evidence based on in vivo and in vitro studies that caffeine has some positive effects in AD models, but further studies are warranted to identify all the mechanistic pathways of the neuroprotective effect of caffeine in AD. 

## Data Availability

Not applicable.
